# All-*trans* retinoic acid inhibits glioblastoma progression and attenuates radiation-induced brain injury

**DOI:** 10.1172/jci.insight.179530

**Published:** 2024-11-08

**Authors:** Min Fu, Yiling Zhang, Bi Peng, Na Luo, Yuanyuan Zhang, Wenjun Zhu, Feng Yang, Ziqi Chen, Qiang Zhang, Qianxia Li, Xin Chen, Yuanhui Liu, Guoxian Long, Guangyuan Hu, Xiaohong Peng

**Affiliations:** 1 Department of Oncology, Tongji Hospital, Tongji Medical College, Huazhong University of Science and Technology, Wuhan, China.; 2 Department of Radiology, The First Affiliated Hospital, Zhejiang University School of Medicine, Hangzhou, China.

**Keywords:** Aging, Brain cancer, Cell cycle

## Abstract

Radiotherapy (RT) remains a primary treatment modality for glioblastoma (GBM), but it induces cellular senescence and is strongly implicated in GBM progression and RT-related injury. Recently, eliminating senescent cells has emerged as a promising strategy for treating cancer and for mitigating radiation-induced brain injury (RBI). Here, we investigated the impact of all-*trans* retinoic acid (RA) on radiation-induced senescence. The findings of this study revealed that RA effectively eliminated astrocytes, which are particularly prone to senescence after radiation, and that the removal of senescence-associated secretory phenotype factor–producing astrocytes inhibited GBM cell proliferation in vitro. Moreover, RA-mediated clearance of senescent cells improved survival in GBM-bearing mice and alleviated radiation-induced cognitive impairment. Through RNA sequencing, we found that the AKT/mTOR/PPARγ/Plin4 signaling pathway is involved in RA-mediated clearance of senescent cells. In summary, these results suggest that RA could be a potential senolytic drug for preventing GBM progression and improving RBI.

## Abstract 2

RA could be a potential senolytic drug for inhibiting GBM progression and improving RBI.

## Graphical abstract

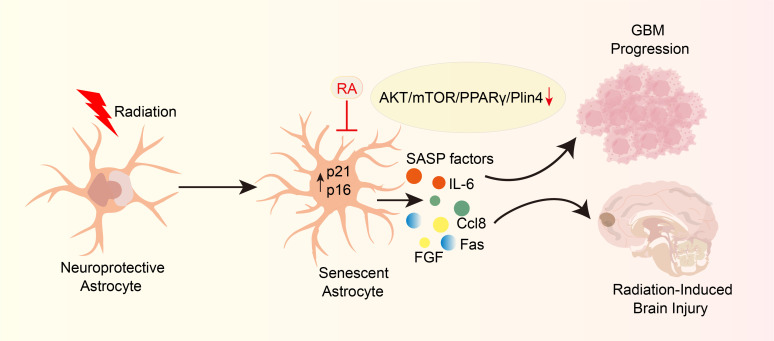

## Introduction

Glioblastoma (GBM) is the most common and aggressive brain tumor in adults, and the median age of GBM onset of 68 to 70 years (1). The median survival time of patients with GBM is approximately 12–16 months (2). Radiotherapy (RT) is the primary approach for managing GBM; however, 80% of recurrence cases occur at the resection margin where the highest doses of radiation are administered (3–5). In addition, RT causes injury to healthy brain tissues and results in central nervous system (CNS) complications (6, 7); this condition is known as radiation-induced brain injury (RBI), which manifests as cognitive impairment and brain tissue necrosis and seriously impacts the quality of life of patients with GBM (7, 8). Hence, the identification of strategies that attenuate the growth of GBM cells and minimize neurotoxicity after RT is urgently needed (9). Recent research has revealed the potential of senolytic drugs to address not only GBM progression, but also RBI (10–15).

Cellular senescence is a state of permanent cell cycle arrest that is regulated by the p53/p21 (CIP1) and/or p16INK4A/Rb pathways. It is characterized by relative resistance to apoptosis, persistent DNA damage signaling, changes in heterochromatin structure, reduced lamin B1 levels, enhanced senescence-associated β-galactosidase (SA-β-gal) activity, and the acquisition of a senescence-associated secretory phenotype (SASP) (16). In the context of cancer, various stressors, such as DNA damage, oncogene activation, exposure to therapeutic agents, and elevated reactive oxygen species (ROS), can trigger cellular senescence (17).

Radiation is a stress factor that is capable of inducing cellular senescence (18). In the context of tumorigenesis, senescence can exert dual effects, and its outcomes are determined by the specific context (19). Persistently senescent cells secrete growth factors, interleukins, extracellular matrix (ECM) components, and ECM-modifying metalloproteases that together result in a tumor-promoting SASP (20, 21). Notably, the partial elimination of senescent cells that express high levels of p16INK4A (hereafter referred to as p16) improves the survival of GBM-bearing mice (10). These findings suggest that senolytic drug therapy could serve as a valuable adjunctive treatment for patients with GBM. Moreover, senolytic therapy might also help mitigate the neurodegeneration and cognitive deficits caused by RT in patients with brain tumors.

RBI may cause progressive cognitive deterioration, which manifests as dementia-like symptoms (7); additionally, this condition shares pathological characteristics with aging-associated neurodegeneration, including excessive glial cell activation, neuroinflammation, blood-brain barrier (BBB) destruction, demyelination, and reduced neurogenesis (18, 19). Radiation-induced cellular senescence is recognized as an important mediator of tissue dysfunction, and it promotes chronic inflammation and contributes to brain injury. It has been demonstrated that astrocytes preferentially undergo senescence after radiation (12), and senescent astrocytes release SASP factors, such as TNF-α, IL-1β, and IL-6, which participate in tissue injury and impair neurogenesis in the CNS (20, 21). Pharmacological or genetic depletion of senescent astrocytes has been demonstrated to promote neuroprotection and improve cognition (12, 14, 22). Therefore, inhibiting radiation-induced cellular senescence–mediated neuroinflammation has emerged as a promising therapeutic approach for treating RBI.

All-*trans* retinoic acid (RA), which is an active metabolite of vitamin A, can effectively penetrate the BBB (23–25), and it plays a crucial role in CNS development, cellular differentiation, and homeostasis (26). Jaeckle et al. documented that temozolomide (TMZ) and 13-*cis*-retinoic acid (cRA) have shown activity in recurrent malignant gliomas without overlapping toxicity in phase II clinical trials (27). See et al. (28) demonstrated the efficacy and durability of cRA for some patients, but did not confirm the better results reported in the phase II study (29), a discrepancy that could be due to the small number of patients enrolled in that study. Consequently, the evidence supporting the use of RA for glioma treatment remains limited. Moreover, previous studies have shown that a low concentration (1 nM) of RA extends the lifespan of normal human epidermal keratinocytes (NHEKs) in vitro by stimulating proliferation and delaying senescence (30, 31). Given the possible effects of RA on senescence, in this study, we sought to investigate whether RA could prevent the progression of GBM and improve RBI. We established models of radiation-induced senescence in vitro and in vivo, and we evaluated the phenotypic changes that were associated with senescent cells and assessed the impact of RA on these changes. Our findings demonstrated that RA increases the survival of tumor-bearing mice and ameliorates neurocognitive impairment by eliminating radiation-induced senescent cells. These results suggest a potential therapeutic role for RA as a senolytic drug in the prevention of GBM progression and the attenuation of RBI.

## Results

### RA eliminates radiation-induced senescent astrocytes in vitro.

**Figure 1:**
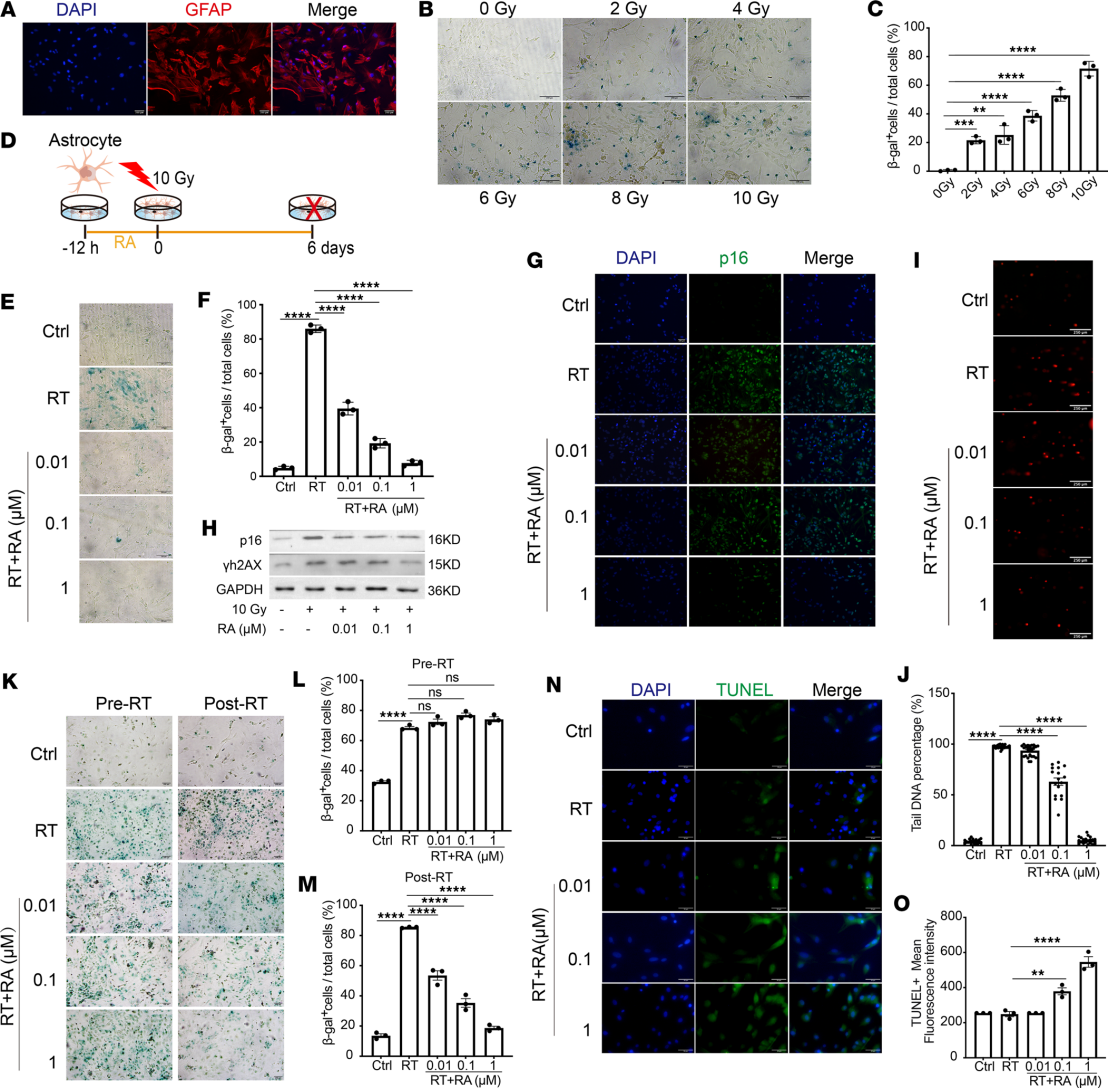
RA eliminates senescent mouse astrocytes after radiation in vitro. (**A**) Primary mouse astrocytes were stained with DAPI (blue) and for GFAP (red). Representative images are shown in the figure. (**B**) Plot shows representative SA-β-gal staining pictures of astrocytes irradiated at different doses. (**C**) Plot shows β-gal^+^ cells relative to the total number of cells; the β-gal^+^ cells were correlated with radiation doses. (**D**) Schematic diagram of the in vitro experiments. (**E**) Detection of senescent astrocytes by SA-β-gal staining. Plot shows representative SA-β-gal images of senescent astrocytes. Ctrl, control. (**F**) Plot shows the percentage of SA-β-gal^+^ astrocytes in the total cells. (**G**) Fluorescent staining of senescent astrocytes after 6 days of irradiation with 10 Gy. Images show immunofluorescent staining of senescent astrocytes for p16 (green) and with DAPI (blue). (**H**) Western blot of astrocytes (10 Gy, 6 days) for p16 and γh2AX. (**I** and **J**) Cells were harvested and analyzed using the alkaline comet assay (**I**), and a total of approximately 100 cells per condition are shown (**J**). (**K**) Plot showing representative images of SA-β-gal staining of astrocytes treated with RA before or after radiation. (**L** and **M**) Plot showing the number of β-gal^+^ cells relative to the total number of cells. RA was administered prior to radiation (**L**), and RA was administered after radiation (**M**). (**N** and **O**) Apoptosis in astrocytes was assessed through TUNEL immunofluorescent staining. (**N**) Nuclear DNA (blue) was stained with DAPI. (**O**) The mean fluorescence intensity of TUNEL staining is shown. Individual dots represent replicate wells (*n* = 3 replicate wells per condition). Data are presented as mean ± SEM and were analyzed by 1-way ANOVA (**C**) or 2-way ANOVA (**F**, **J**, **L**, **M**, and **O**) with Tukey’s multiple-comparison test. **P < 0.01; ***P < 0.001; ****P < 0.0001. Scale bars: 200 μm (**A**, **B**, **E**, **G**, and **K**), 250 μm (**I**), and 50 μm (**N**).

A previous study identified astrocytes as the major senescent cell type in irradiated brains (12). Primary astrocytes were isolated from neonatal mouse or rat brains and identified by the expression of glial fibrillary acidic protein (GFAP; ref. 12) (Figure 1A). We first treated astrocytes with different radiation doses (0, 2, 4, 6, 8, or 10 Gy), and the results showed that RT induced astrocyte senescence in a dose-dependent manner (Figure 1, B and C). Therefore, 10 Gy was selected as the appropriate dose for RT-induced astrocyte senescence. Then, we investigated whether RA could eliminate senescent astrocytes in vitro. Various concentrations of RA (32) were administered to astrocytes 12 hours before irradiation for 6 days (12) (Figure 1D). Irradiated astrocytes that were treated with RA exhibited reduced SA-β-gal activity, particularly after treatment with higher concentrations of RA (1 μM) (Figure 1, E and F). Immunofluorescence and protein expression analyses further revealed that p16 and γh2AX protein expression was decreased in RA-treated astrocytes compared with irradiated astrocytes, which was consistent with impaired SA-β-gal activity (Figure 1, G and H). A similar pattern was observed in primary astrocytes that were harvested from rats (33), which were characterized by the absence of SA-β-gal^+^, p16^+^, and γh2AX^+^ cells after RA treatment (30, 31, 34) (Supplemental Figure 1, A–E; supplemental material available online with this article; https://doi.org/10.1172/jci.insight.179530DS1). DNA damage is a key hallmark of senescence (35). Therefore, we conducted comet electrophoresis to detect the effect of RA on DNA damage in astrocytes after irradiation. The degree of comet tailing in the irradiated cells treated with RA was significantly lower than that in the RT group, indicating that RA could reduce radiation-induced DNA damage (Figure 1, I and J). In addition, to further elucidate whether RA inhibited senescence induction or functioned as a senolytic agent, we treated astrocytes with RA before or after irradiation, respectively. The findings demonstrated that RA administered following radiation reduced SA-β-gal activity, while RA administered prior to radiotherapy did not result in a decrease in the number of β-gal^+^ cells (Figure 1, K–M). These findings support the ability of RA to postpone, but not prevent, senescence (30). Many studies suggest that senescent cells exploit antiapoptotic machinery to survive (36). Next, we aimed to investigate the effect of RA on the apoptosis in senescent astrocytes. TUNEL staining showed that RA significantly increased the number of TUNEL-positive astrocytes compared with that in the irradiated group (Figure 1, N and O). Taken together, these results indicate that RA can eliminate radiation-induced senescent astrocytes and promote their apoptosis.

### Elimination of the SASP by RA inhibits the migration of GBM cells.

**Figure 2:**
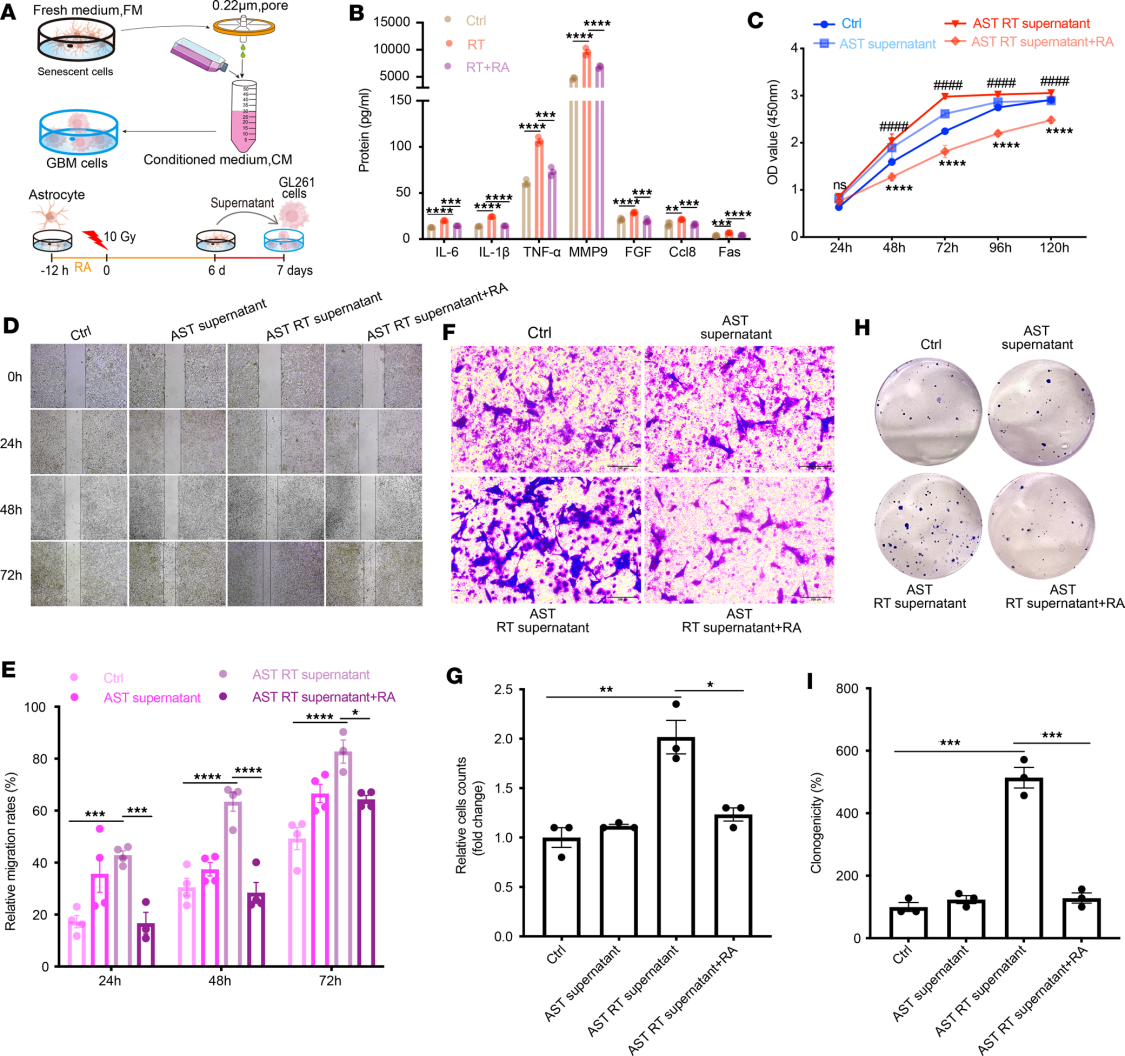
RA inhibits SASP-induced GL261 cell proliferation and migration. (**A**) Schematic diagram of GL261 cells incubated with radiation-induced senescent astrocyte supernatants. The supernatants of astrocytes 6 days after 10 Gy irradiation were centrifuged and filtered with 0.22-μm filters, and then added to GL261 cells and cultured for 24 hours. (**B**) ELISA analysis of IL-6, IL-1β, TNF-α, MMP9, FGF, Ccl8, and Fas protein levels in the irradiated astrocyte culture supernatant. (**C**) Cell viability at different time points in the control (Ctrl), astrocyte supernatant (AST supernatant), supernatant of irradiated astrocytes (AST RT supernatant), and RA with supernatants of irradiated astrocytes (AST RT supernatant+RA). ^####^Indicates comparison with the Ctrl group; ****indicates comparison with the AST RT supernatant group. (**D**) Scratch-wound assay results of GL261 cells from various groups at different time points. Scale bar: 250 μm. (**E**) Images from panel **D** were quantified using ImageJ software. (**F** and **G**) Transwell experiments were used to assess the invasion of GL261 cells incubated with supernatants. (**F**) The images show GL261 cell invasion after supernatant treatment. Scale bars: 200 μm. (**G**) The graph shows relative invasive cell counts (relative to Ctrl); an average cell count of 3 fields was randomly taken under the ×20 objective. (**H**) Colony formation of GL261 cells after supernatant treatment. The panel shows GL261 cell proliferation after supernatant treatment. (**I**) Graph shows the percentage of clone formation. Data are presented as mean ± SEM and were analyzed by 2-way ANOVA with Tukey’s multiple-comparison test. NS, not significant. *P < 0.05; **P < 0.01; ***P < 0.001; ****P < 0.0001; ^####^*P* < 0.0001.

Accumulating evidence highlights the pivotal role of SASP factors in driving cancer cell invasion and maintaining a cancer stem cell phenotype in GBM (37). To assess the effect of RA on SASP-mediated GBM cell growth, we treated GL261 or C6 cells with conditioned media (CM) from radiation-induced senescent astrocytes that were treated with or without RA (Figure 2A). An analysis of these supernatants revealed that the cytokine profile of senescent astrocyte supernatants included a significant increase in the levels of pro-oncogenic SASP factors, including IL-6, IL-1β, TNF-α, MMP9, FGF, Ccl8, and Fas. Importantly, RA treatment significantly blocked the radiation-induced production of these SASP factors in culture (Figure 2B). To better understand the effect of the SASP on GBM cell behavior, a CCK-8 assay was used to evaluate the impact of senescent astrocytes on the viability of GBM cells. Compared with incubation with CM collected from the RT-treated astrocytes, CM collected from astrocytes that were treated RA significantly inhibited glioma cell viability, as expected (Figure 2C). Wound-healing and Transwell assays are typically used to evaluate tumor cell migration in vitro, and tumor cell migration is closely related to tumor staging and clinical outcome. We found that CM collected from irradiated astrocytes significantly promoted the migration of GL261 cells in Transwell migration and wound-healing assays, whereas CM collected from nonirradiated astrocytes had no such effect; furthermore, incubation with RA-treated astrocyte–derived CM resulted in a significant decrease in GL261 cell migration across the Transwell membrane and a significant increase in average wound width (Figure 2, D–G). Moreover, consistent with the changes in the CCK-8 assay, the addition of CM collected from irradiated astrocytes accelerated cell colony formation, whereas the percentage of colony formation was decreased when CM was derived from RA-treated astrocytes (Figure 2, H and I). Furthermore, these in vitro results were verified in another GBM cell line, namely C6 cells, and the results showed that the SASP factors that were secreted by RT-induced senescent astrocytes promoted the proliferation and migration of C6 cells. In contrast, cell survival was significantly decreased after incubation with the CM derived from RA-treated astrocytes (11, 30, 38) (Supplemental Figure 2, A–H). Together, our findings confirm the inhibitory effects of RA on SASP-mediated GBM cell proliferation and migration in an in vitro model.

### Partial removal of senescent cells with RA increases the survival of GBM-bearing mice.

**Figure 3:**
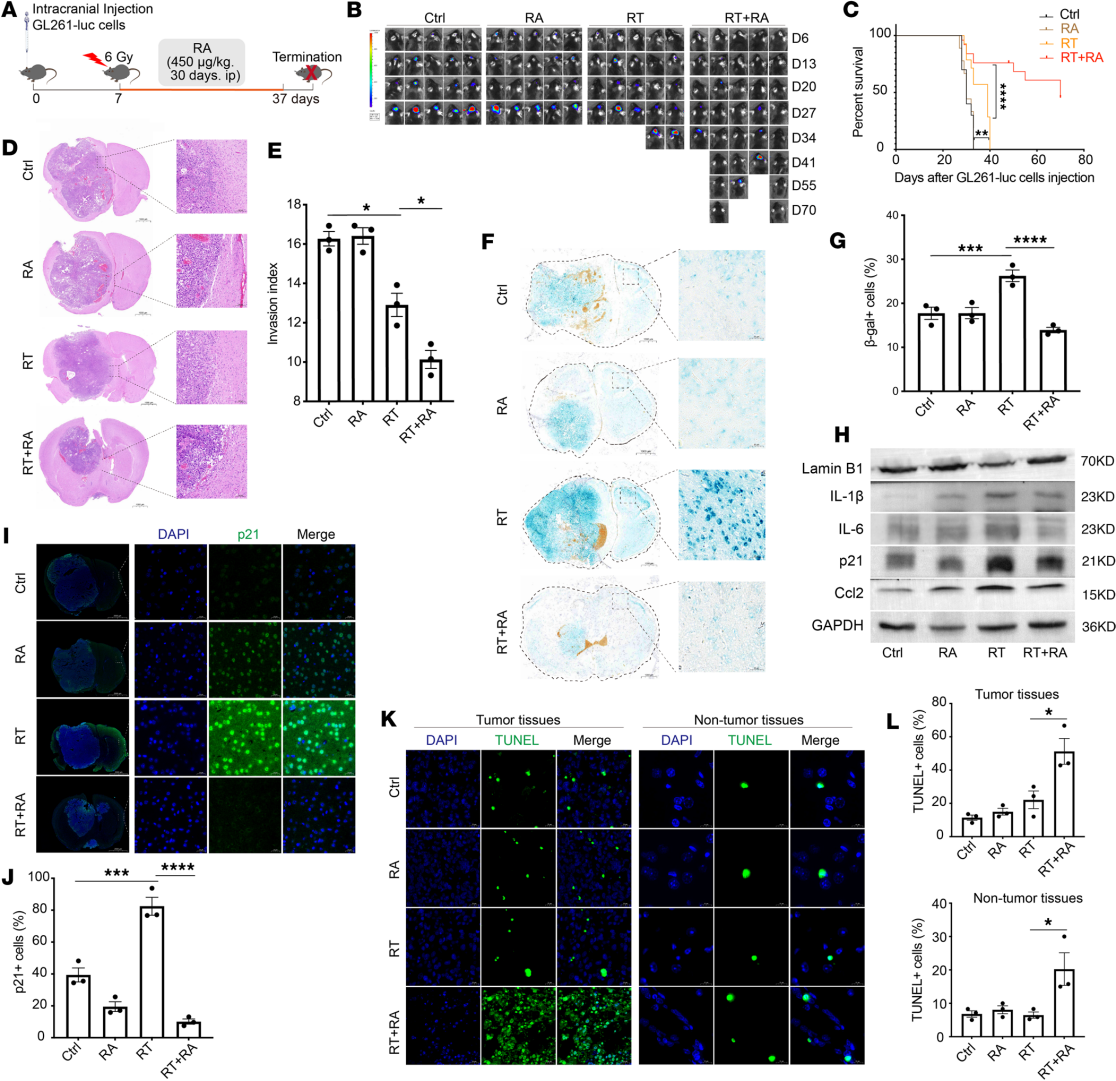
RA reduces senescent cells and extends the survival of GBM mice. (**A**) Schematic of experimental design. (**B**) Representative BLI images showing GL261-luc tumor growth in individual mice from the different treatment groups. Ctrl, control. (**C**) Kaplan-Meier survival curves for mice bearing intracranial GL261-luc tumors. Median survival for mice treated with RT+RA was 55 days versus 39 days for RT alone. (**D**) H&E-stained brain sections from mice bearing intracranial GBM. High magnifications show representative areas spanning the tumor borders and tumor regions. Note that treatment with RA had significantly reduced brain tumor size and infiltration. Scale bars: 100 μm. (**E**) Plot depicts average invasive index in different groups. (**F**) Representative high-magnification images of SA-β-gal staining in brain tumor. Scale bars: 50 μm. (**G**) Quantification of the percentage of β-gal^+^ cells in mice. (**H**) Western blot images of lamin B1, IL-1β, IL-6, p21, and Ccl2. (**I**) Images show immunofluorescent staining of GBM mouse brains for p21 (green) and with DAPI (blue). Right panels represent higher magnifications of the left panels. Scale bars: 20 μm. (**J**) Plot shows the percentage of p21^+^ cells in GBM mouse brains. (**K** and **L**) TUNEL staining showing the effect of RA treatment on the apoptosis of tumor and non-tumor cells. (**K**) Representative TUNEL images are shown. Scale bars: 20 μm (left panels) and 10 μm (right panels). (**L**) The quantification of the percentage of TUNEL-positive cells is shown. *n* = 10–20 mice per group for survival analyses; *n* = 4–6 mice per group for Western blotting analysis, H&E, and SA-β-gal staining; *n* = 3 mice per group for immunofluorescent staining. Data are presented as mean ± SEM and were analyzed by 2-way ANOVA with Tukey’s multiple-comparison test. *P < 0.05; **P < 0.01; ***P < 0.001; ****P < 0.0001.

To investigate whether RA may delay GBM progression by eliminating senescent cells, orthotopic implantation of GL261-luc cells into the brains of mice was performed, followed by monitoring of tumor growth using bioluminescence imaging (BLI). Seven days after implantation (39), the mice were treated with DMSO (control), RA alone (450 μg/kg/d), local RT (6 Gy) (40), or RT plus RA (RT+RA) (41) (for the treatment schedule, see Figure 3A). At 27 days, the BLI signals were significantly lower in the mice receiving RT+RA treatment than in those receiving only RT or RA alone, reflecting a large difference in tumor size (Figure 3B). The median survival of mice bearing intracranial GL261-luc tumors treated with the combination of RT and RA was significantly longer than that of mice treated with RT alone (Figure 3C). Histopathological analyses revealed a clear response to RA treatment, evidenced by markedly reduced tumor growth and invasion (Figure 3, D and E). Many studies suggest that senescent cells could represent an actionable target to mitigate the process of gliomagenesis (10, 42, 43). To investigate whether RA’s antitumor effects are associated with a reduction in radiation-induced senescent cells, we performed SA-β-gal staining to elucidate RA’s impact on senescence in GBM mice. Compared with irradiated mice, GBM-bearing mice that were treated with RT+RA had significantly fewer β-gal^+^ cells in both tumor and nontumor tissues (Figure 3, F and G). Consistent with this, by protein and immunofluorescence analysis, we observed that, compared with irradiated mice, RA significantly reduced the expression levels of p21, IL-1β, IL-6, and Ccl2, while upregulating lamin B1 (Figure 3, H–J). Additionally, we explored the effects of RA on senescent GBM cells in vitro and found that RA effectively eliminated RT-induced senescent GBM cells (Supplemental Figure 3, A–F). Interestingly, the number of TUNEL-positive cells in both tumor and nontumor cells increased following RA treatment (Figure 3, K and L). Collectively, these data suggest that RA could reduce senescent cells, and extend the survival of tumor-bearing mice.

### RA alleviates cognitive impairment and ameliorates RBI.

**Figure 4:**
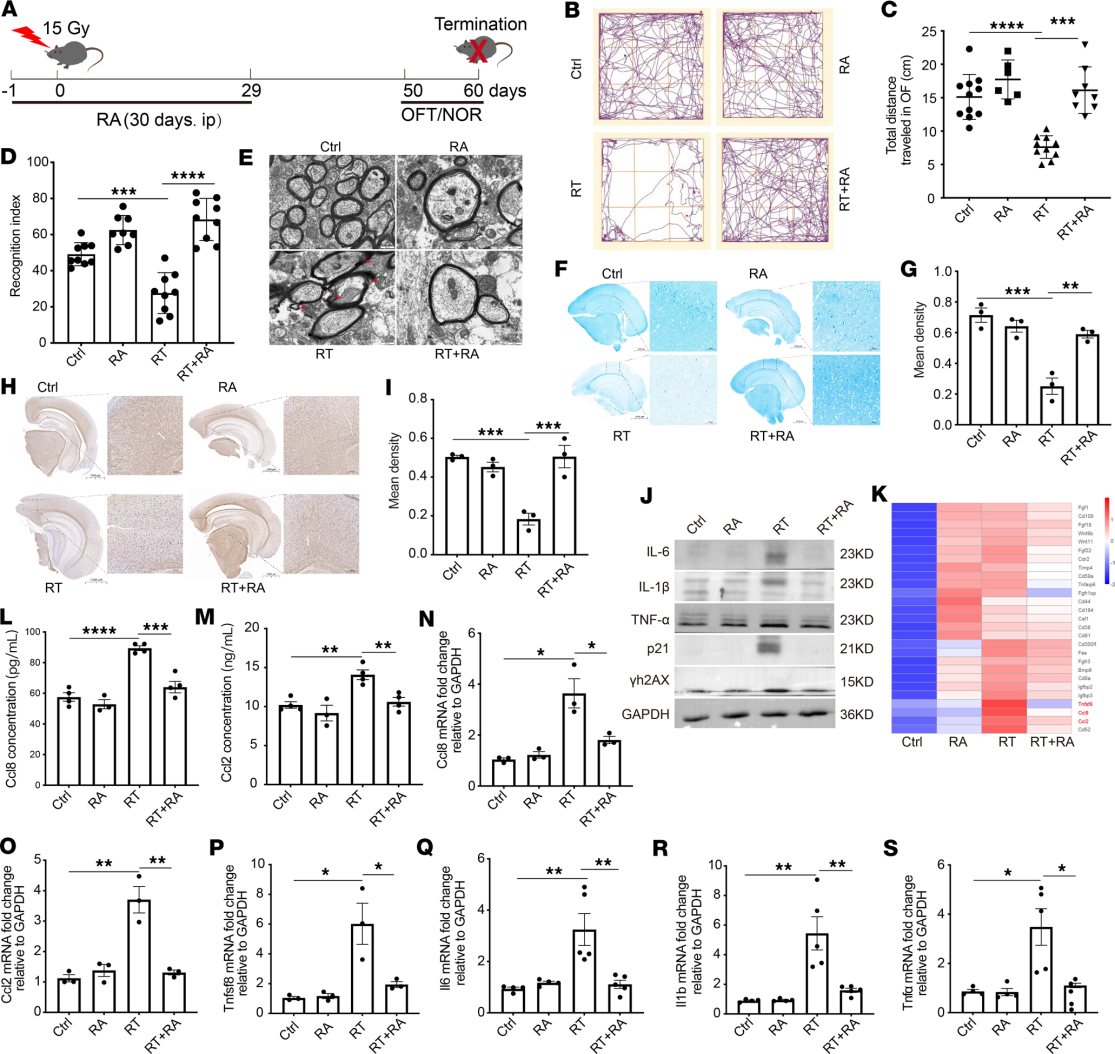
RA alleviates cognitive impairment and ameliorates RBI. (**A**) Timeline of the RA intervention and tests. (**B** and **C**) The movement trajectory (**B**) and total distance traveled (**C**) of mice from different groups in the OFT. Ctrl, control. (**D**) Plot represents recognition index of new objects of mice from different groups in the NOR test. (**E**) Representative TEM images of sections from mice in different groups. Scale bars: 500 nm. (**F**) Brain sections were stained with Luxol fast blue. Scale bars: 50 μm. (**G**) The mean intensity of the cortex in the different groups was analyzed. (**H**) The image shows the areas analyzed by performing immunocytochemical staining for MBP in the cortex of mice. Scale bars: 100 μm. (**I**) Graphs showing the intensity of MBP staining in the cortex. (**J**) Western blotting for senescence markers after 60 days. (**K**) RNA-seq analysis of genes (27) exhibiting significant changes in expression after irradiation that were rescued by RA inhibition. (**L** and **M**) ELISA analysis of Ccl8 and Ccl2 protein levels in radiated brain. (**N**–**S**) qRT-PCR analysis of SASP gene mRNA levels in brain after radiation. *n* = 8–9 mice per group for NOR and OFT analyses; *n* = 4–6 mice per group for Western blotting, ELISA, and qRT-PCR analyses; *n* = 3 mice per group for the RNA-seq and LFB and IHC staining. Each dot represents 1 mouse. Data are presented as mean ± SEM and were analyzed by 2-way ANOVA with Tukey’s multiple-comparison test. *P < 0.05; **P < 0.01; ***P < 0.001; ****P < 0.0001. Four groups were established for in vivo study: Ctrl, control mice treated with 450 μg/kg (RA), irradiated mice (RT), and irradiated mice treated with 450 μg/kg RA (RT+RA).

To explore the potential protective effects of RA on a mouse model of RBI induced by a single 15-Gy dose of radiation, we considered the RA dose that was used in other neurodegenerative studies (44). Ultimately, low, medium and high doses of RA (150 μg/kg/d, 450 μg/kg/d, and 900 μg/kg/d, respectively) were used in this study (Figure 4A). Daily monitoring of mouse body weight after RA treatment revealed that only the medium dose of RA (450 μg/kg/d) had a protective effect on weight loss in irradiated mice (Supplemental Figure 4A). The open-field test (OFT) results revealed that mice that were treated with a medium dose (450 μg/kg/d) of RA traveled a greater distance (cm), spent more time (s) in the central area, spent less time immobile (s), and crossed the platform more times than mice that were exposed to radiation alone (Figure 4, B and C, and Supplemental Figure 4, B–I). Moreover, the novel object recognition (NOR) test was used to evaluate cognitive status, and the results indicated a greater recognition index and preference for exploring the novel object in the RA group (450 μg/kg/d) than in the group treated with radiation alone (Figure 4D and Supplemental Figure 4, J–L). In summary, changes in performance in these behavioral tests showed that cognitive memory disorders caused by radiation were significantly ameliorated by the administration of RA. Based on these results, we selected a medium dose of RA (450 μg/kg/d) for further analysis. Radiation-induced damage to the BBB is recognized as the pathological basis of RBI (45). Intercellular tight junctions (TJs) play a crucial role in BBB formation. We further explored the impact of RA on TJ proteins in irradiated brains. In comparison with those in the control group, the expression levels of ZO-1 and VE-cadherin were significantly decreased in irradiated mice; these effects were strongly reversed by RA treatment (Supplemental Figure 4, M–O). The irradiated brain exhibits significant pathological demyelination (46). TEM analysis of the cortex also revealed that radiation led to demyelination, and treatment with RA appeared to reduce the demyelination caused by RT (Figure 4E). We next applied Luxol fast blue (LFB) staining and immunohistochemistry to assess the severity of myelin injury. LFB staining revealed larger lesions in irradiated mice than in RA-treated mice (Figure 4, F and G). Consistent with the LFB staining results, the immunohistochemical analysis revealed lower expression of myelin basic protein (MBP) in mice after radiation, and treatment with RA increased the protein expression of MBP compared with that in the irradiated group (Figure 4, H and I). Astrocyte senescence and astrocyte-derived neuroinflammation have been identified as potential contributors to RBI (12). We aimed to understand the molecular mechanisms underlying the beneficial effects of RA on RBI. Brain tissues were collected and changes in proinflammatory factor levels were evaluated. The protein levels of proinflammatory factors, including IL-6*,* IL-1β, and TNF-α, as well as senescence-associated factors, including p21 and γh2AX, were found to be increased after irradiation, which was consistent with previously published reports (20, 47). Importantly, RA treatment significantly reduced the expression of these cytokines (Figure 4J and Supplemental Figure 4, P–T). RNA-seq revealed an increase in the expression of numerous genes associated with the SASP (Figure 4K), and we used ELISA and qRT-PCR to validate these changes in gene expression. The data showed that *Ccl8*, *Ccl2*, *Tnfsf8*, *Il6*, *Il1b*, and *Tnfa* were significantly upregulated after radiation compared with the control group, and their expression was decreased in RA-treated mice (Figure 4, L–S). Taken together, these results indicate that RA exerted significant pharmacological effects on reducing senescence and inhibiting the inflammatory response, suggesting that RA might potentially be useful for alleviating RBI.

### The AKT/mTOR/PPARγ/Plin4 signaling pathway is involved in RA-mediated clearance of senescent cells.

**Figure 5:**
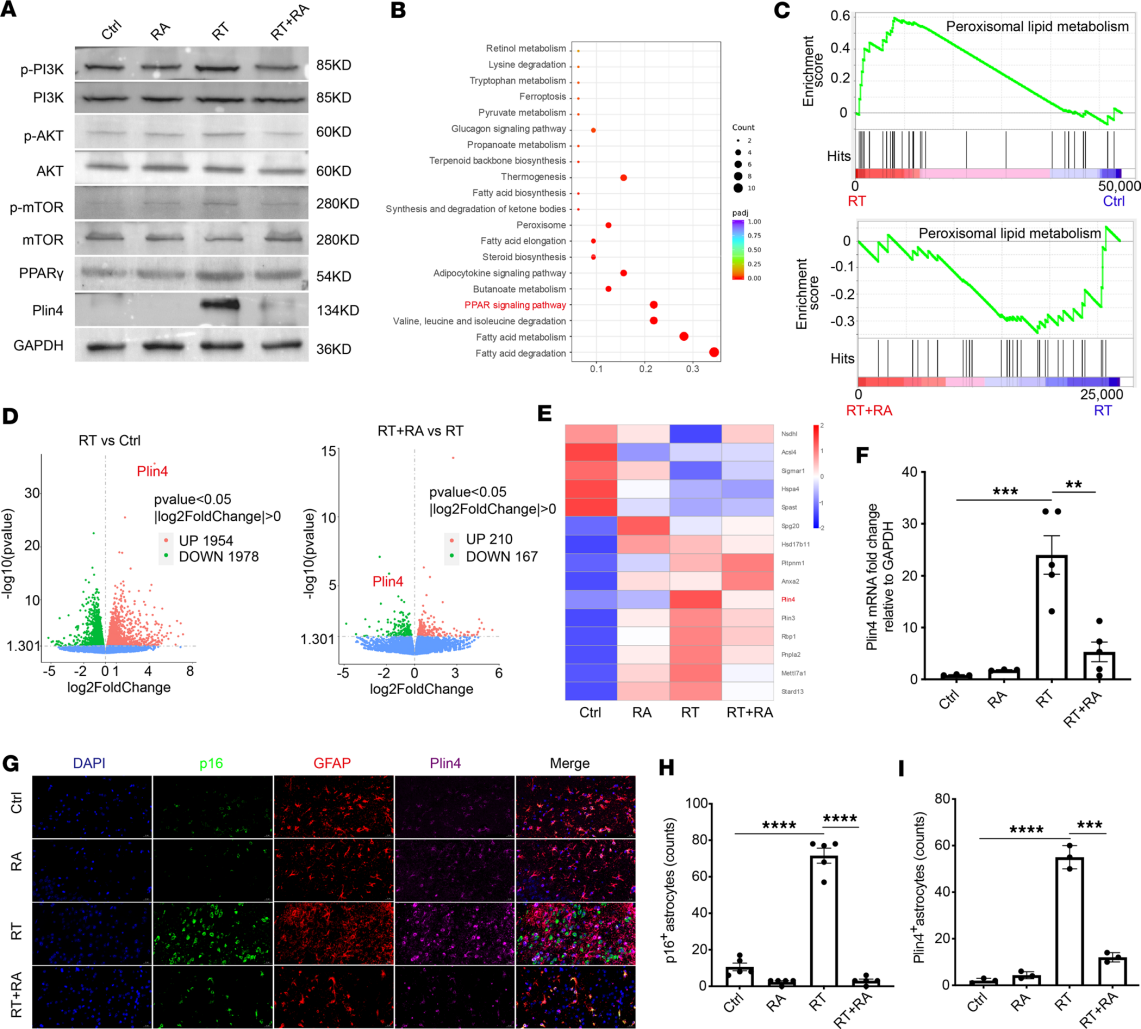
The AKT/mTOR/PPARγ/Plin4 signaling pathway is implicated in the RA-induced elimination of senescent cells. (**A**) Western blotting of PI3K/AKT/mTOR/PPARγ/Plin4 pathway activation. Ctrl, control. (**B**) KEGG pathway analysis of differentially expressed genes. (**C**) Enrichment plots from GSEA. GSEA results suggest that the peroxisomal lipid metabolism response pathways are upregulated after radiation. (**D**) The significantly upregulated and downregulated genes identified in the transcriptome analysis of the brain tissues from the control group, the irradiated group, and the RA intervention group. (**E**) Heatmap of differential expression of lipid-related genes in mouse brain tissues. (**F**) qRT-PCR analysis of *Plin4* mRNA levels in the brain of RBI mice after RA treatment. (**G**) Representative images of p16 (green), GFAP (red), and Plin4 (pink) colabeling in the brain of RBI mice. Scale bars: 20 μm. (**H** and **I**) Quantification of the number of GFAP^+^ astrocytes positive for p16 (**H**) and Plin4 (**I**). *n* = 4–6 mice per group for Western blotting and qRT-PCR analyses; *n* = 3 mice per group for the RNA-seq and immunofluorescent staining. Data are presented as mean ± SEM and were analyzed by 2-way ANOVA with Tukey’s multiple-comparison test. **P < 0.01; ***P < 0.001; ****P < 0.0001.

Studies have demonstrated that sustained hyperactivation of the PI3K/AKT/mTOR pathway in nontransformed cells triggers cellular senescence (48); these results are consistent with our finding that RT causes constitutive PI3K/AKT/mTOR pathway activation and increases cellular senescence (Figure 5A and Supplemental Figure 5, A–C). Additionally, the AKT/mTOR/PPARγ pathway is known to regulate lipid synthesis and uptake (49, 50). We observed that irradiation was accompanied by significant increases in p-AKT, p-mTOR, and PPARγ, but these levels were decreased by RA treatment (Figure 5A and Supplemental Figure 5, D and E). These results suggested that RA inhibited radiation-induced activation of AKT/mTOR/PPARγ signaling. To further confirm the effects of RA-mediated senescent cell elimination on the AKT/mTOR/PPARγ pathway, we conducted RNA-seq analyses on RNA that was extracted from brain tissues. Kyoto Encyclopedia of Genes and Genomes (KEGG) analysis revealed that the genes that were upregulated after irradiation were involved mainly in fatty acid metabolism and the PPAR signaling pathway (Figure 5B). Gene set enrichment analysis (GSEA) of the RNA-seq data revealed the upregulation of peroxisomal lipid metabolism in irradiated mice compared with that in control or RA-treated mice (Figure 5C); these results were consistent with the increased PPARγ expression that was observed by Western blotting (Supplemental Figure 5F). To further understand the downstream targets of the AKT/mTOR/PPARγ pathway, we performed a comprehensive analysis of the transcriptome data. The results revealed that 1954 genes were significantly upregulated in the radiation group compared with the control group, whereas 1978 genes were significantly downregulated. Compared with those in the radiation group, 210 genes were upregulated in the RA intervention group, whereas 167 genes were downregulated (Figure 5D). Focusing on the genes whose expression was changed in response to radiation and subsequently restored by RA treatment, the most significantly upregulated gene, namely, *Plin4*, was selected for further exploration (Figure 5E). We observed that *Plin4* mRNA expression was upregulated by 21.68-fold after radiation (Figure 5F), and protein analysis confirmed this finding (Figure 5A and Supplemental Figure 5G). Immunofluorescent costaining revealed that p16 and Plin4 colocalized with GFAP^+^ cells in irradiated brains but not in the brains of RA-treated mice (Figure 5, G–I). In vitro experiments also revealed that the majority of Plin4 colocalized with p16^+^ astrocytes, indicating that senescent astrocytes were the predominant source of Plin4 (Supplemental Figure 5, H and I). Collectively, these data indicate that theAKT/mTOR/PPARγ/Plin4 signaling pathway plays a role in the RA-induced elimination of senescent cells.

## Discussion

In this study, we revealed what we believe is a new application of RA; that is, we showed that RA can eliminate senescent cells, delay GBM progression, and ameliorate cognitive dysfunction. We further confirmed that the AKT/mTOR/PPARγ/Plin4 pathway is crucial for RA-mediated clearance of senescent cells through in vivo and in vitro experiments.

Increasing evidence suggests the potential benefits of senolytic drug therapy for GBM (10, 11). In our specific study, we found that clearance of senescent cells by RA resulted in slower growth of the implanted glioma cells and improved survival of the tumor-bearing mice. To further investigate the impact of the SASP on GBM cells, we conducted in vitro experiments using senescent astrocyte–derived supernatants to stimulate GBM cells. The results demonstrated that the proliferation of GBM cells was substantially inhibited when they were exposed to supernatants completely derived from senescent cells. These findings suggest that the composition of the SASP is dynamically and spatially regulated and that changes in the composition of the SASP can determine the beneficial and detrimental aspects of the senescence program, tipping the balance toward either a tumor-suppressive environment or a tumor-promoting state (51); these results emphasize the importance of the timing of senolytic drug administration after cancer therapy. Remarkably, senolytic drugs are most effective when administered in a hit-and-run fashion, minimizing potential toxic and off-target effects.

Cellular senescence is recognized as an important mediator of tissue dysfunction, promoting chronic inflammation and contributing to radiation-induced side effects, including RBI (52, 53). Although neurons are terminally differentiated cells, Herdy et al. reported that they can still exhibit features of senescence under prolonged stress (54). This senescence is typically accompanied by the release of SASP, which further activate nearby cells, including astrocytes and microglia, leading to chronic inflammatory responses (54). In our study, we similarly observed that radiation triggered widespread senescence in mouse brains. By coimmunostaining mouse brain sections for GFAP and senescence markers, we found that mouse brain astrocytes were particularly prone to senescence. This finding is consistent with previous studies showing that astrocytes compose the bulk of senescent cells in irradiated human and mouse brains (12). Therefore, we focused on primary astrocytes to verify the inhibitory effect of RA on senescent cells in vitro. The results indicated that following RA treatment, there was a substantial reduction in radiation-induced senescent astrocytes, accompanied by an increase in TUNEL-positive apoptotic cells. Through the combination of in vitro and in vivo studies, we found that RA can downregulate SASP-related gene expression by clearing senescent cells, mitigate the inflammatory response, and increase the levels of ZO-1 and VE-cadherin (55), effectively in reducing myelin loss. Consequently, RA has the potential to attenuate cognitive dysfunction and ameliorate RBI.

Numerous studies have demonstrated that persistent activation of the PI3K/AKT/mTOR pathway promotes a senescent phenotype that is associated with various senescence markers (48, 56). Consistent with these findings, our results revealed that RA eliminated radiation-induced senescence, which was accompanied by inhibition of the PI3K/AKT/mTOR signaling pathway. Another critical feature that is associated with RA-mediated clearance of senescent cells is a marked change in metabolic activity, characterized by decreased lipid contents compared with those in senescent cells. RNA-seq analyses revealed the suppression of the AKT/mTOR/PPARγ pathway after RA treatment, concomitant with decreased expression of lipid-related genes such as *Plin4*, *Plin3*, *Rbp1*, *Pnpla2*, *Mettl7a1*, and *Stard13* (57). Among these genes, the PPARγ target gene *Plin4* was most markedly upregulated after radiation (58). These findings highlight that the AKT/mTOR/PPARγ/Plin4 pathway is involved in RA-mediated clearance of senescent cells. Overall, these effects of RA, a well-established therapeutic agent, provide evidence for the preclinical application of senolytic drugs, and these findings may accelerate the translation of such drugs in clinical trials.

As a therapeutic drug, RA plays a crucial role in gliomas and neurodegenerative diseases, such as stroke and Alzheimer disease (59, 60), demonstrating great potential value in clinical applications. The results from the phase II trial suggest that the combination of TMZ and cRA may be a more active regimen for recurrent malignant gliomas (27). Yung et al. conducted a phase II prospective study of 43 patients with recurrent malignant glioma treated with cRA as a single agent, and a response rate of 23% was observed (29). In addition, the responses to cRA reported in the retrospective review (28) are modest, but compared with the responses to conventional cytotoxic agents used to treat patients with recurrent GBM (61), cRA is orally available and well tolerated, with side effects consisting mainly of hypercholesterolemia, hypertriglyceridemia, and skin rash (28). RA plays diverse roles in malignant conditions. We hope that clinical trials combining retinoids with cytotoxic agents, as well as immunotherapy, will reveal the potential for the synergism and tolerability of multiple agents with different antitumor mechanisms.

However, due to poor water solubility and short half-life of RA, it is challenging to administer, and fine-tuning the concentration range is needed to achieve its effects (62). We found that nonirradiated mice that were treated with RA at high (900 μg/kg/d) or low (150 μg/kg/d) doses exhibited substantial decreases in weight, activity level, and ability to recognize new objects, suggesting that an abnormal level of RA can have deleterious effects on mice (63, 64). Notably, a previous study indicated that treating mice with a clinical dose (1 mg/kg/d) of RA severely disrupts the capacity to learn a spatial radial maze task (65). In addition, Min et al. reported that NHEKs exhibited the characteristics of replicative senescence when treated with greater than 1 μM RA (30). Another study showed that increasing concentrations of RA (1–10 μM) effectively induced cellular senescence in human cancer cells (66). Hence, delivery of a therapeutic dose is critical for the effective elimination of senescent cells by RA. Of course, choosing the time point for RA treatment is also a key to reducing senescence; our findings indicate that the preventive use of RA does not effectively eliminate senescence. Therefore, we prefer to use RA as a senolytic agent to eliminate senescent cells rather than prevent senescence induction.

This study has several limitations. Firstly, we did not examine all of the multiple pathways that are potential mediators of cell survival and tumor progression. Numerous studies have shown that the Wnt/β-catenin pathway is vital to the pathophysiology of malignant glioma and linked to malignant glioma stem cell proliferation, invasiveness, and treatment resistance (67). Furthermore, EGFR/STAT3 and Notch signaling support self-renewal and proliferation of GBM stem cells to drive tumor progression (68, 69). Future studies are warranted to further identify the key pathways that are involved in regulation of cell survival and perform studies to explore the translational potential of blocking these signaling pathways. Secondly, our study focused on the role of RA in the clearance of senescent cells. Recent evidence suggests that ionizing radiation–induced senescence greatly impairs immune cell functions in mice (70), whereas elimination of senescent cells restores immune homeostasis within the tumor microenvironment and increases mouse survival in response to immunotherapy (71). However, our current studies did not investigate the changes in immune cell function after the elimination of senescent cells by RA. In addition, we have not determined whether these changes in immune cells contribute to the observed phenotype. Future studies should explore whether the use of RA could be a pharmacological approach to improve the effectiveness of cancer immunotherapies.

RA, which has controlled toxicity and is affordable, appears to be more promising for clinical translation than other senolytic drugs, such as ABT263 (navitoclax), quercetin, and dasatinib. ABT263, which is an inhibitor of the BCL-xL family, has known toxic side effects, including transient thrombocytopenia and neutropenia (72). The second-generation BCR-ABL tyrosine kinase inhibitor dasatinib (73), compared with imatinib and nilotinib, has increased potency and CNS penetration (74). However, it can also cause overall myelosuppression, such as transient and reversible platelet deficiency, neutropenia, and anemia (75, 76). The limited spectrum of senescent cells that are targeted by BCL-xL inhibitors alone further restricts their efficacy (77, 78). These adverse effects pose challenges for antisenescence therapies, especially those requiring prolonged treatment intervals (79). Thus, the translation of senolytic drugs to the clinic has been challenging. In contrast, RA is a repurposed anticancer agent with known on- and off-target effects. While its dose and scheduling must be optimized for clinical applications, its potential controlled toxicity and affordability render it a more favorable candidate for translation. Further investigations are imperative to fine-tune dosing cycles and identify optimal doses in order to ensure the effective clinical application of RA. Future research could explore strategies to integrate RA senolytic therapies into aggressive anticancer regimens, aiming to delay tumor progression while reducing late complications. In addition, Herdy et al. highlight the importance of neuronal senescence in brain aging and disease progression (54), suggesting that future studies could further explore the role of RA in neuronal senescence and its potential application in other neurodegenerative diseases.

## Methods

### Sex as a biological variable.

Male mice were used in the experiment because male mice are more likely than female mice to activate microglia under irradiation conditions, which leads to greater brain injury in male mice (80–83). Mice (C57BL/6J background) were randomly assigned to all analyses.

### Animal studies.

C57BL/6J mice (male, 6–8 weeks) were purchased from Hunan SJA Laboratory Animal and raised in a specific pathogen–free laboratory. Sprague-Dawley (SD) rats (P0–P1) were obtained from Biont Biological Technology. Mice were anesthetized with 1.5% pentobarbital sodium and received a 15-Gy x-ray dose to the head (anterior–posterior) using the RS2000 x-ray Biological Research Irradiator (160 kV, 25 mA, Source Technologies Inc.). Control mice were anesthetized and sham irradiated. For intracranial stereotactic injections, we selected a murine orthotopic GL261 GBM model, because tumors established from GL261 cells recapitulate many characteristics of human GBMs and are commonly used in studies of glioma immunotherapy and in preclinical studies (84). Luciferase-tagged GL261 cells (100,000 cells) were suspended in PBS (1 μL) and injected into the right corpus striatum of the brains of C57BL/6J mice. Focal radiation (6 Gy) was applied to the brain of tumor-bearing mice 7 days after implantation of GL261-luc cells (40). Tumor development was monitored by BLI; mice were injected with D-luciferin potassium (40902, Yeasen) and anesthetized with 1.5% pentobarbital sodium. Bioluminescence was detected using the IVIS 200 Xenogen system (IVIS Spectrum, PerkinElmer).

### Drug administration.

RA (HY-14649, MedChemExpress) was diluted to final concentration of 3 mg/mL in normal saline (0.9% NaCl), which was prepared freshly at the time of injection. RA was injected intraperitoneally at 10:00 am every day for 30 consecutive days at a dose of 150 μg/kg, 450 μg/kg, and 900 μg/kg body weight (44), respectively, starting from the day before the mice were irradiated. The mice were injected at a total volume of 5 μL per gram body weight and the saline-only injection was used in the nontreated control groups. The body weight of mice was recorded daily. For cellular administration, RA was dissolved in dimethylsulfoxide (DMSO) and then diluted with an appropriate volume of medium to yield different concentrations of RA working solution (32) (from 0.01 μM to 1 μM).

### Alkaline comet assay.

Primary astrocytes were trypsinized, and 3000 cells per condition were embedded in 75 μL of low-melting point agarose (KTA3040, Abbkine) on 3-well comet slides (100 μL per well) and lysed for 1 hour at 4°C in lysis buffer (2.5 M NaCl, 500 mM EDTA, 10× Lysis Solution, pH 10), and incubated for 30 minutes in alkaline solution (NaOH and 500 mM EDTA), followed by electrophoresis for 20 minutes at 30 V and 200 mA. Afterward, all slides were neutralized with 0.4 mM Tris-HCl solution at 4°C for 10 minutes. DNA staining was performed with 1× PI dye for 10 minutes at room temperature and protected from light, followed by washing with PBS. Samples were imaged using a microscope (EVOS FL Auto, Thermo Fisher Scientific) and analyzed using OpenComet v1.3 (https://cometbio.org/documentation.html).

### Apoptosis assay.

Using a terminal deoxynucleotidyl transferase (TdT) dUTP Nick-End Labeling (TUNEL) staining to analyze the level of apoptosis. The astrocytes were washed with PBS and fixed with 4% paraformaldehyde for 20 minutes. After the specimens were washed with PBS 3 times, 0.3% Triton X-100 was used to permeabilize the cells for 30 minutes, and then they were stained with TUNEL working solution (KTA2010, Abbkine) in accordance with the protocol. The nuclei were counterstained with 1× DAPI for another 10 minutes. Lastly, a fluorescence microscope was used to analyze the fluorescence signals.

### Primary astrocyte isolation and cell culture.

Primary astrocytes were derived from mixed glial cultures using the “shaking off” method. Briefly, the brain stem, meninges, and cerebellum from neonatal (P0–P1) C57BL/6J mouse or SD rat were removed in ice-cold HBSS buffer (MA0039, Meilunbio), and the remaining parts were cut into 1-cm^3^ pieces and digested using 0.25% trypsin-EDTA (25200056, Gibco) at 37°C for 15 minutes and fresh medium was used to stop digestion. After filtration and centrifugation, cells were collected and cultured in medium for glial cells and then cultured in a 75-cm^2^ cell culture flask coated with poly-D-lysine (10 mg/mL; Sigma-Aldrich) at 37°C in a humidified 5% CO_2_ incubator. The medium was changed every 3 days for a total culture time of 10–14 days. Astrocytes were shaken off from the primary glial cell culture (150 rpm, 37°C, overnight) between days 10 and 14. The floating cells were collected and seeded into poly-D-lysine–pretreated 6-cm dishes and incubated at 37°C in a humidified 5% CO_2_ incubator. Primary astrocytes and GL261 and C6 cells were cultured in DMEM (SC102-02, Seven) supplemented with penicillin and streptomycin (KGL2304-100, Keygen BioTECH) and 10% fetal bovine serum (FBS-CS500, Newzerum). C6 is a rat glioma cell line that is histopathologically classified as an astrocytoma cell line and a previous study revealed that the changes in gene expression observed in the C6 cell line were the most similar to those reported in human brain tumors (85). Therefore, it represents a widely used model for studying human GBMs (86). GL261 cells are p53 mutant and K-ras mutant (87), whereas C6 glioma cells are p53 wild-type and p16 mutant (88, 89). All cells were mycoplasma free, and early-passage cells were used for experiments.

### Western blotting.

Brain tissue– and cell-derived proteins were extracted using RIPA buffer (P0013B, Beyotime) supplemented with phenylmethylsulfonyl fluoride (G2008-1M, Servicebio) and phosphatase inhibitor cocktail (HY-K0021, MedChemExpress), and the proteins were solubilized using sodium dodecyl sulfate polyacrylamide gel electrophoresis (SDS-PAGE) loading buffer (P1041, Solarbio). Immunoblotting was performed using a Bio-Rad gel system. The proteins were resolved in 10% SDS-PAGE gels, followed by transfer of the proteins to 0.45-μm polyvinylidene fluoride (PVDF) membranes (IPVH00010, Millipore). After blocking with 5% BSA for 1 hour, the membranes were incubated with primary antibodies against the following proteins: GAPDH (1:20,000; 60004-1-lg, Proteintech), α-tubulin (1:20,000; 66031-1-Ig, Proteintech), IL-1β (1:1000, A1112, ABclonal), IL-6 (1:2000; 21865-1-AP, Proteintech), TNF-α (1:1000, 17590-1-AP, Proteintech), ZO-1 (1:1000; A0659, ABclonal), VE-cadherin (1:1000; A0734, ABclonal), p21 (1:500; sc-6246, Santa Cruz Biotechnology), p16 (1:500; sc-1661, Santa Cruz Biotechnology), lamin B1 (1:1000; A16909, ABclonal), γh2AX (1:1000; AP0687, ABclonal), p-mTOR (1:1000; AP0094, ABclonal), mTOR (1:1000; A2445, ABclonal), p-PI3K (1:1000; AP0854, ABclonal), PI3K (1:1000; A22487, ABclonal), p-AKT (1:1000; T40067, Abmart), AKT (1:1000; T55561, Abmart), PPARγ (1:1000; sc-7273, Santa Cruz Biotechnology), Plin4 (1:1000; bs17031R, Bioss), and Ccl2 (1:1000; YP-Ab-11161, UpingBio) at 4°C overnight. The next day, the membranes were incubated with secondary antibodies for 1 hour at room temperature. Subsequently, the immunoblots were analyzed using Super Signal ECL Chemiluminescent Substrate (P10100, New Cell & Molecular Biotech) in the G: BOX Chemi X system (Syngene) and analyzed using ImageJ software (NIH).

### ELISA.

Primary astrocytes isolated from brains of neonatal mice and rats were cultured in 6-cm dishes. A single dose of 10 Gy x-ray radiation effectively induced senescence and was used in this study (12); cell media were replaced with complete culture media and treated with different concentrations of RA (from 0.01 μM to 1 μM) 12 hours before irradiation. Six days later, the culture supernatant was collected for ELISA analysis to detect the levels of TNF-α, IL-1β, IL-6, MMP9, Ccl8, FGF, and Fas. According to the manufacturer’s instructions (Ruixinbio Quanzhou), 50 μL of standard, control, or samples was added to a 96-well plate, and then 100 μL of horseradish peroxidase–conjugated detection antibody was added to each well and incubated for 1 hour in the dark. After washing 5 times, 100 μL of 3,3′,5,5′-tetramethylbenzidine (TMB) substrate was added to each well for 15 minutes. Finally, 50 μL of stop solution was added and the absorbance at 450 nm was determined using a microplate reader (BioTek).

### Statistics.

All statistical analyses and graph generation were performed using GraphPad Prism 9. A 2-tailed Student’s *t* test was performed to analyze data between 2 experimental groups, while multiple group comparisons were performed using 1-way analysis of variance (ANOVA). A repeated measures 2-way ANOVA was applied to compare data from different groups in different time points. Kaplan-Meier functions were used to illustrate survival profiles using the Mantel-Cox test. A *P* value of less than 0.05 was considered significant. NS, not significant.

### Study approval.

The animal experiments were performed in accordance with the guidelines of the International Guiding Principles for Animal Research and approved by the Ethics Committee of Huazhong University of Science and Technology, located in Wuhan, Hubei, China (permit no. TJH-202104017).

### Data availability.

Values for all data points in graphs are reported in the Supporting Data Values file. The data and materials that support the findings of this study are available from the corresponding author upon reasonable request.

## Author contributions

MF was responsible for formal analysis, validation, investigation, writing the original draft, and reviewing and editing the manuscript. Yiling Zhang and BP contributed to reviewing and editing the manuscript. NL, Yuanyuan Zhang, WZ, FY, ZC, and QZ carried out formal analysis, validation, and investigation. QL, XC, YL, and GL were responsible for formal analysis. GH and XP were responsible for conceptualization, formal analysis, supervision, and funding acquisition.

## Supplementary Material

Supplemental data

Unedited blot and gel images

Supporting data values

## Version 1 11/08/2024

Electronic publication
