# Supplementary material for: All-trans retinoic acid inhibits glioblastoma progression and attenuates radiation-induced brain injury
Source: JCI Insight. 2024 Nov 8;9(21):e179530. doi: 10.1172/jci.insight.179530 (PMC11601587; doi:10.1172/jci.insight.179530)
Supplement: Supplemental data [file jciinsight-9-179530-s019.pdf]

Supplemental Figure

Supplemental Figure 1

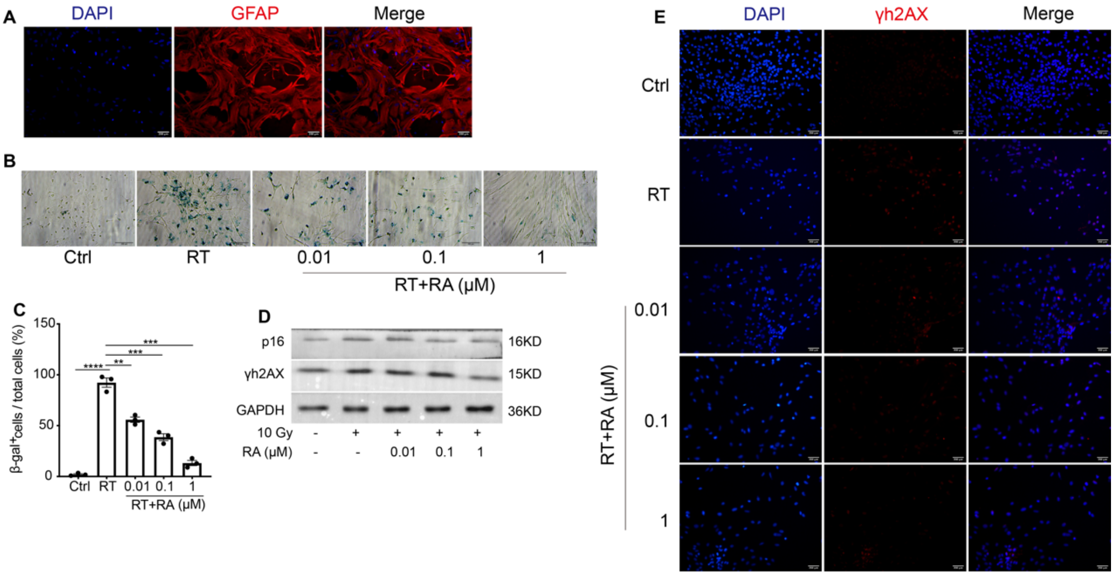

RA reduces senescence of rat astrocytes after radiation in vitro.

(**A**) Primary rat astrocytes were stained for DAPI (blue) and GFAP (red). Representative images are shown in the figure. Scale bar, 200 μm. (**B**) Detection of senescent astrocytes by SA-β-gal staining, plot shows representative SA-β-gal images of senescent astrocytes. Scale bar, 200 μm. (**C**) Plot shows β-gal+ astrocytes relative to the total number of cells, irradiated astrocytes experienced a significant, dose-dependent decrease in SA-β-gal staining after RA treatment. (**D**) Western Blot of radiated astrocytes (10 Gy, 6 days) for p16 and γh2AX. (**E**) Images show immunofluorescence staining of astrocytes for DAPI (blue) and γh2AX (red). Scale bar, 200 μm. Data are presented as means ± SEM and analyzed by 2-way ANOVA. $**P< 0.01$, $***P < 0.001$, $****P < 0.0001$.

Supplemental Figure 2

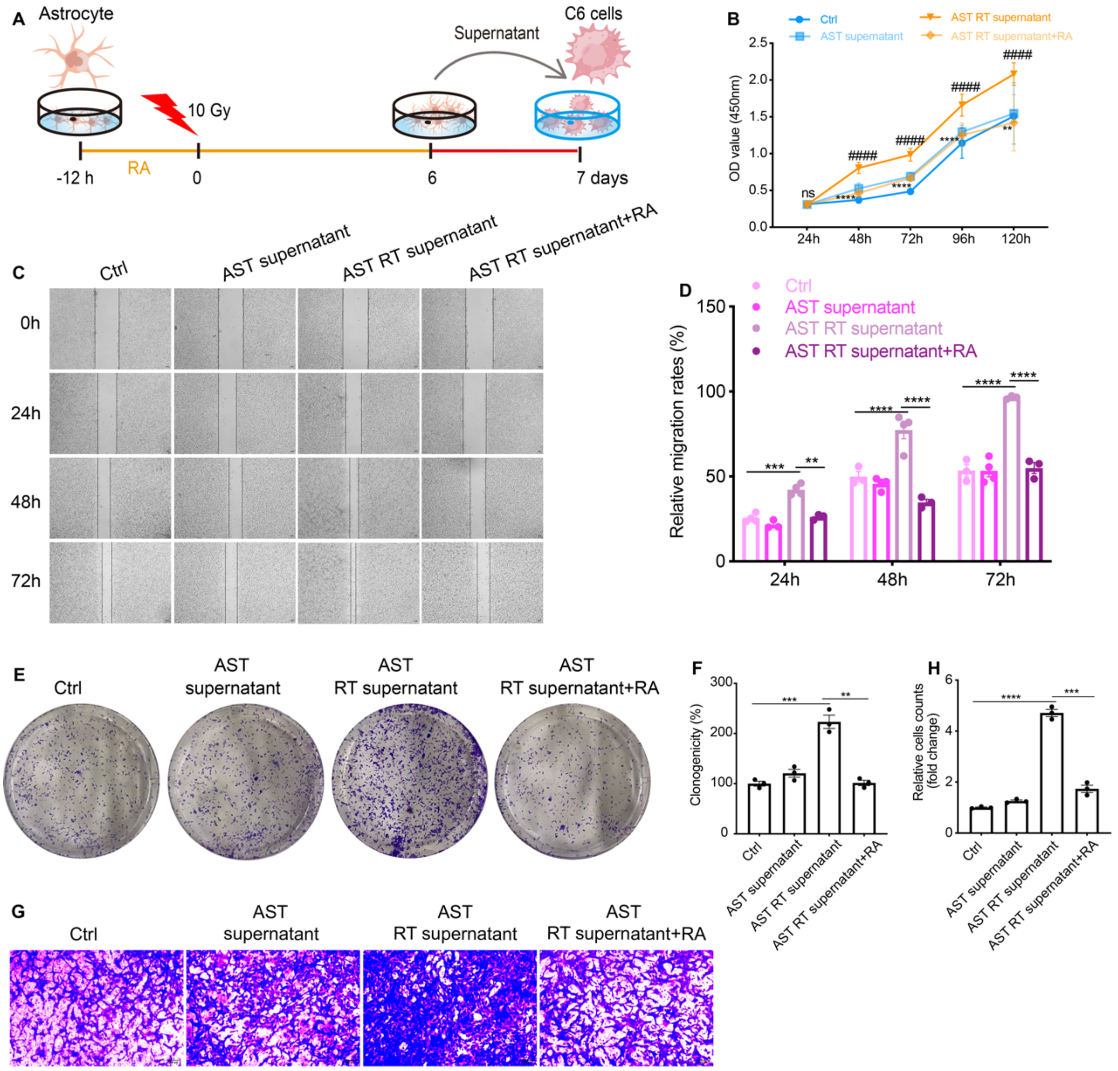

RA inhibits SASP-induced C6 cells proliferation and migration.

(**A**) Diagram illustrates the coculture of C6 cells with the irradiated-induced senescent astrocytes supernatant. (**B**) Cell viability at different time points in the control, astrocytes supernatant (AST supernatant), supernatant of irradiated astrocytes (AST RT supernatant), RA with supernatants of irradiated astrocytes (AST RT supernatant+RA). #: vs Ctrl group; ∗: vs AST RT supernatant group. (**C** and **D**) Wound healing assays were used to assess the migration of C6 cells treated with supernatants for 24 h, 48 h and 72 h. The left panel shows C6 cells migration after supernatants treatment. The right panel shows the cells migration rates. Scale bar, 250 μm. (**E**) Colony formation of C6 cells after supernatants treatment. The panel shows C6 cells proliferation after supernatants treatment. (**F**) Graph shows the clones formation of C6 cells treated with supernatants (relative to Ctrl). (**G** and **H**) Transwell experiments were used to assess the invasion of C6 cells incubated with supernatants. The left panel shows C6 cells invasion after supernatants treatment. Scale bar, 200 μm. The graph on the right shows relative invasive cell count (relative to Ctrl). Data are presented as means ± SEM and analyzed by 2-way ANOVA. ns, not significant, $*P < 0.05$, $**P < 0.01$, $***P < 0.001$, $****P < 0.0001$.

Supplemental Figure 3

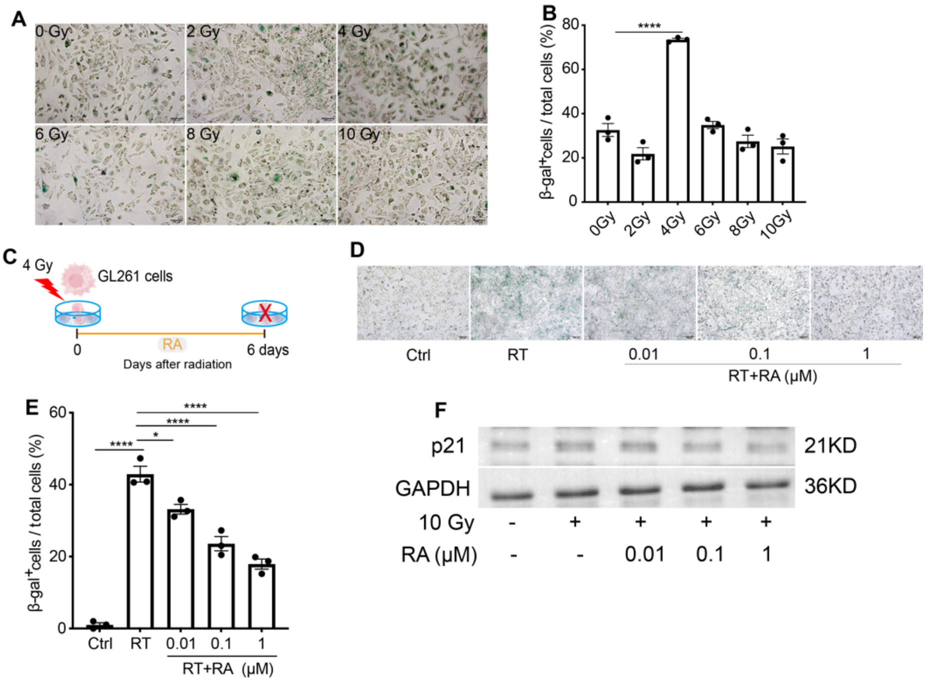

RA reduces radiation-induced senescent GL261 cells.

(**A**) Plot showing representative images of SA-β-gal staining in GL261 cells irradiated at different doses. Scale bar, 200 μm. (**B**) Plot showing β-gal+ cells relative to the total number of cells. (**C**) Schematic diagram of GL261 cells were treated with RA. (**D**) Detection of senescent glioma cells using SA-β-gal staining. The plot shows representative images of SA-β-gal staining in senescent GL261 cells. Scale bar, 200 μm. (**E**) Plot showing the percentage of SA-β-gal+ GBM cells to the total cells. (**F**) Western blot showing p21 levels in GL261 cells (4 Gy, 6 days). Data are presented as means ± SEM and analyzed by one-way ANOVA (B) or 2-way ANOVA (E). $*P < 0.05$, $**P < 0.01$, $***P < 0.001$, $****P < 0.0001$.

Supplemental Fig.4

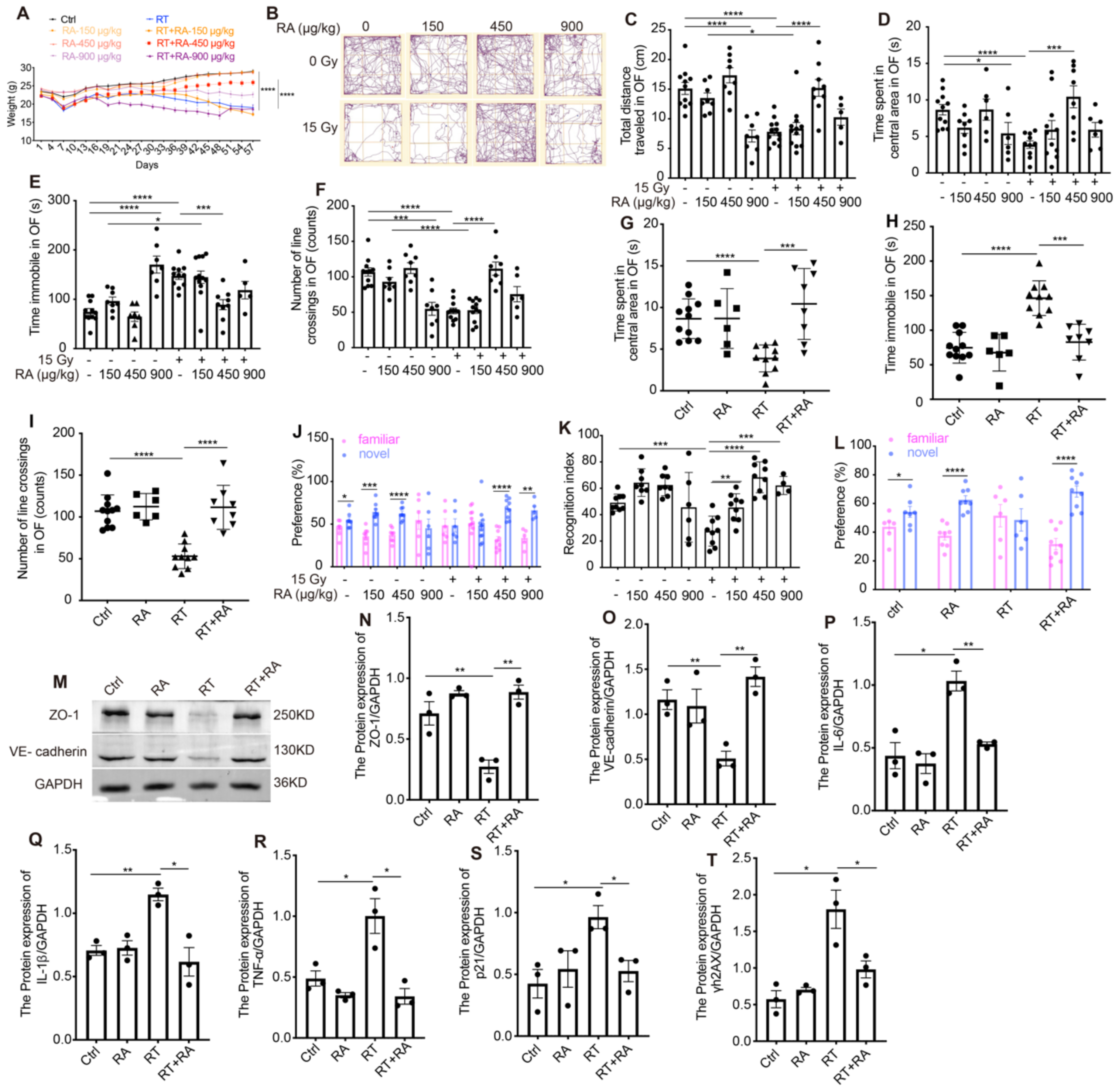

RA attenuates cognitive dysfunction and ameliorates RBI.

(**A**) Plot shows body weight changes in mice from irradiation to death. (**B-F**) The traveled trajectory (**B**), distance (**C**), time spent in central (**D**), immobile time (**E**) and number of line crossings (**F**) of mice from eight groups for 10 min in the OFT. (**G-I**) time spent in central (**G**), immobile time (**H**) and number of line crossings (**I**) of mice from four groups in the OFT. (**J**) Plot depicts percentage preference for familiar and novel objects in eight groups of mice in the NOR test. (**K**) Recognition index of novel objects in eight groups of mice in the NOR test. (**L**) Percentage preference for familiar and novel objects in four groups of mice in the NOR test. (**M**) Western Blot of mice brain tissues (15 Gy, 60 days) for ZO-1, VE-cadherin, GAPDH was probed as loading control. (**N-T**) quantification of ZO-1(**N**), VE-cadherin (**O**), IL6 (**P**), IL1B (**Q**), TNFA (**R**), p21 (**S**) and γh2AX (**T**) by ImageJ software. Data are presented as means ± SEM and analyzed by 2-way ANOVA. $*P< 0.05$, $**P< 0.01$, $***P < 0.001$, $****P < 0.0001$. Eight groups were established for in vivo study: control mice (Ctrl), control mice treated with RA (RA-150 μg/kg, RA-450 μg/kg and RA-900 μg/kg), irradiated mice (RT), and irradiated mice treated with RA (RT+RA-150 μg/kg, RT+RA-450 μg/kg and RT+ RA-900 μg/kg). Four groups as previously described.

Supplementary Fig.5

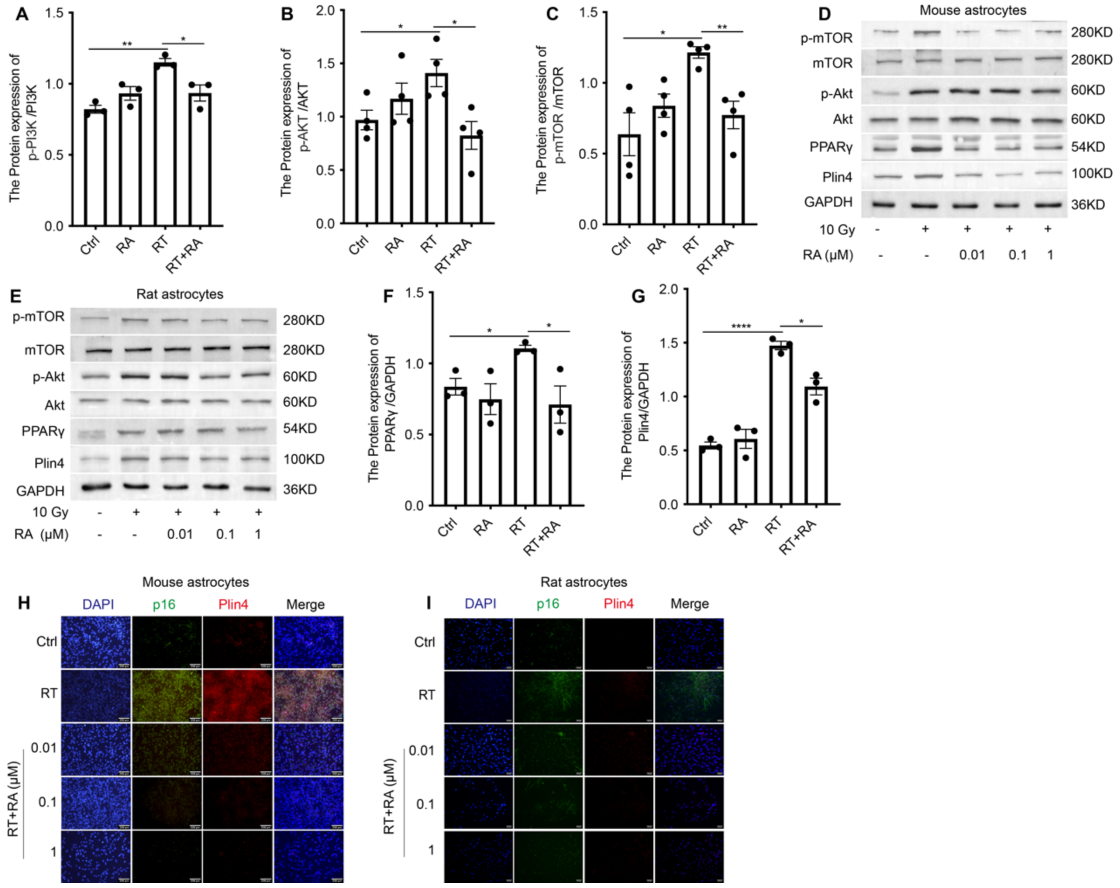

The AKT/mTOR/PPARγ/Plin4 signaling pathway is associated with the RA-mediated clearance of senescent cells.

(**A-C**) quantification of p-PI3K (**A**), p-AKT (**B**), p-mTOR (**C**), by ImageJ software. (**D** and **E**) Western blotting of AKT/mTOR/PPARγ/Plin4 pathway activation in irradiated primary mouse astrocytes (**D**) and rat astrocytes (**E**). (**F** and **G**) quantification of PPARγ (**F**), Plin4 (**G**) by ImageJ software. (**H** and **I**) Fluorescent staining of astrocytes 6 days after 10 Gy irradiation, images show immunofluorescence staining of irradiated primary mouse astrocytes (**H**) and rat astrocytes (**I**) for DAPI (blue), p16 (green) and Plin4 (red). Scale bar, 200 μm. Data are presented as means ± SEM and analyzed by 2-way ANOVA. *$P< 0.05$, **$P< 0.01$, ****$P < 0.0001$.

Supplemental Table 1. Primer sequences used in qRT-PCR.

Primer	Sequence (5’-3’)
GAPDH forward	CATGGCCTTCCGTGTTCCTA
GAPDH reverse	CCTGCTTCACCACCTTCTTGA
Il1b forward	GCAACTGTTCCTGAACTCAACT
Il1b reverse	ATCTTTTGGGGTCCGTCAACT
Il6 forward	CTGCAAGAGACTTCCATCCAG
Il6 reverse	AGTGGTATAGACAGGTCTGTTGG
Tnfα forward	CAGGCGGTGCCTATGTCTC
Tnfα reverse	CGATCACCCCGAAGTTCAGTAG
Ccl8 forward	TCTACGCAGTGCTTCTTTGCC
Ccl8 reverse	AAGGGGGATCTTCAGCTTTAGTA
Ccl2 forward	ATTCTGTGACCATCCCCTCAT
Ccl2 reverse	TGTATGTGCCTCTGAACCCAC
Tnfsf8 forward	GCACAAGTCGCAGCTACTTCT
Tnfsf8 reverse	GGAGTGGAGTCCTTTTTCTGG
Plin4 forward	GTGTCCACCAACTCACAGAT
Plin4 reverse	GGACCATTCCTTTTGCAGCAT

HE and SA-β-gal staining

Mice were perfused with 1× PBS followed by 4% paraformaldehyde (PFA; BP003, Bioss), brains were removed and directly made into frozen sections or immersed in 4% PFA and fixed at 25 °C for 48 h, dehydrated and embedded in paraffin wax to make paraffin sections for hematoxylin and eosin (HE; G1005, Servicebio) and immunofluorescence staining. Frozen sections were stained using the senescence β-galactosidase Staining Kit (KTA3030, Abbkine). Images were captured using a microscope (NIKON, ECLIPSE CI, Japan) equipped with a ×20 objective. 5 fields per well/section were counted and averaged to quantify the percentage of SA-β-gal+ cells.

Luxol fast blue staining (LFB)

Luxol fast blue staining staining was performed to detect demyelinated lesions in brain. After washing in PBS, brain slices were incubated in 0.1% Luxol fast blue (B11003, Baiqiandubio) in acidified 95% ethanol overnight at 60 °C, then cooled and differentiated in Li2CO3 for 2 min. When differentiation was completed, slices were successively dehydrated in 75%, 95% and 100% alcohol and then air-dried. Then slices were mounted by neutral gum (96949-21-2, SCRC). Images were captured using the microscope (NIKON, ECLIPSE CI, Japan), equipped with a ×40 objective. ImageJ was used to calculate the signal intensity. The mean signal intensity of each region per mouse was obtained by initially computing the ratio of the total signal intensity of all visual fields to the total area of the corresponding fields in the indicated region. This process produced the mean signal intensity for the region per section, and these values were then averaged across three non-adjacent sections for each mouse.

Immunocytochemistry (IHC)

Immunohistochemistry was performed on 4 μm thick paraffin-embedded tissues. Tissue sections were deparaffinized in xylene and rehydrated. Endogenous peroxidase activity was quenched at room temperature followed by heat induced epitope retrieval using standard protocols. Slides were immersed in preheated retrieval solution, allowed to cool to room temperature, and then washed with PBS and de-ionized water. Primary antibody incubation was performed using rabbit monoclonal anti-MBP (1:200, A24860, ABclonal) at room temperature for 50 min. Sections were washed with PBS and then incubated with anti- rabbit horseradish peroxidase-conjugated polymer (1:200, 5220-0336, SeraCare) for 50 min at room temperature. The slides were then immersed for 8 min in 25 °C diaminobenzidine (K500711, DAKO), counterstained in hematoxylin, and dehydrated. The brain tissue images were captured using the microscope (NIKON, ECLIPSE CI, Japan) equipped with a ×20 objective. The signal intensity was determined using ImageJ, the mean signal intensity of each mouse was obtained by averaging the signal intensities of three non-adjacent sections.

Immunofluorescence

Formalin-fixed, paraffin-embedded brain sections were depar-affinized, rehydrated, and stained using standard protocols, antigen retrieval was performed at 98℃ for 15 min, in 10 mM/L citrate buffer (pH 6) in a commercial pressure cooker for all antibodies. Tissue sections were blocked in Goat Serum (AR1009, Bosterbio) for 1 h before overnight incubation in primary antibody, then sections were incubated with fluorescent secondary antibodies for 1 h before the nuclei were stained with 4',6-

diamidino-2-phenylindole (DAPI; MA0128, Meilunbio) for 5 min. At last, sections were sealed with anti-quenching fluorescent sealer (SBA-0100-01, SouthernBiotech) and observed under the microscope. The following primary antibodies were used: GFAP (1:200, 16825-1-AP, Proteintech), p16 (1:200, sc-1661, Santa cruz), γh2AX (1:100, AP0687, ABclonal), Plin4 (1:200, bs17031R, Bioss) and p21 (1:200, A19094, ABclonal). For cellular immunofluorescence staining, cells were seeded into 24-well plates before 10 Gy treatment. After 6 days, irradiated cells were fixed with 4% PFA, then permeabilized with 0.5% Triton-100, blocked with 5% Bovine Serum Albumin (BSA; MA4219, Meilunbio), and incubated overnight at 4°C with primary antibody. The next day, the cells were incubated with secondary antibodies for 1 h at room temperature followed by DAPI staining for 5 min. These secondary antibodies were Cy3, goat anti-rabbit IgG (1:500, A22220, Abbkine), DyeLight 488 goat anti-mouse IgG (1:500, A23240, Abbkine). The immunofluorescence images were captured using the microscope (EVOS FL Auto, Thermo Fisher Scientific, Waltham, MA, USA) equipped with a ×40 objective. Immunofluorescence quantification was performed to determine the percentage of positive cells, cells with positive signals enveloping the nucleus or within the nucleus were regarded as positive cells. To calculate the percentage of positive cells in each region per mouse, the procedure involved computing the proportion of positive cells to the total number of cells in the indicated region per section, and these values were averaged across three non-adjacent sections for each mouse.

Transmission electron microscopy (TEM)

The tissues were first fixed with 2.5% glutaraldehyde (Sinopharm Chemical Reagent Co., Ltd) overnight, then washed three times by PBS and postfixed with 1% OsO_4 (20816-12-0, SPI-CHEM) in PBS for 2 h and then rinsed by PBS. The samples were dehydrated gradiently in different concentrations of alcohol for 15 min at each step. Next, the samples were then placed in 1:1 mixture of absolute acetone and the final 812 resin mixture (90529-77-4, SPI) at room temperature, then transferred to the final Spurr resin mixture overnight and heated at 60 °C for 48 h. The samples were sectioned in LEICA EM UC7 ultratome and stained by 2% uranyl acetate and alkaline lead citrate (Sinopharm Chemical Reagent Co., Ltd) for 15 min, respectively, and observed in a JEM-1400Flash TEM.

Open-field test (OFT)

The open field test was conducted in an area consisting of a platform (50 × 50 × 50 cm). The central zone was a square 20 cm away from the wall of the apparatus with a digital camera positioned directly. Each mouse was allowed to explore in the area for 10 min and between each trail the area was cleaned with 75% ethanol. The time spent in the central zone, total travel distance, time immobile and the number of crossings were recorded.

Novel object recognition test (NOR)

The novel object experiment was conducted over two days. On the first day, mice were allowed to explore an open area containing two identical cubes for 10 min. The next day, one of the cubes was replaced by a cylinder in the same position. The animals were allowed to explore the open field for 10 min. Time spent in contacting with objects

was recorded to assess interest in the novel object. Recognition index = time with the cylinder/(time with the cylinder + time with the cube) × 100%.

Quantitative real-time PCR (qRT-PCR)

Brain tissue and cell-derived RNA were lysed with Trizol reagent (NO. 9766, Takara). About 1 μg of total RNA was used for reverse transcription into cDNA by HiScript II Q RT SuperMix (R222-01, Vazyme) in a total volume of 20 μL, qRT-PCR was performed using ChamQ Universal SYBR qPCR Master Mix (Q711-02, Vazyme) in the ABI-7900HT Sequence Quan-tification System (Applied Biosystems, Foster, CA, USA). The mRNA expression levels of the target genes were normalized to GAPDH expression levels and analyzed using the 2-ΔΔCt method. All primers used in this work are listed in Supplemental Table 1.

RNA sequencing

The RNA-seq assay was performed by Novogene (Beijing, China). Briefly, the total RNA of brain tissues was extracted using Trizol and purified for library preparation and sequencing on an Illumina Hiseq platform. Differentially expressed genes (DEGs) were set when the gene expression in the two compared groups was *P* value <0.05. DEGs with the log2FoldChange were used through the R package cluster Profiler for Gene ontology (GO) analysis and Kyoto Encyclopedia of Genes and Genomes (KEGG) pathway, and the results were visualized through the R package ggplot2.

Cell proliferation and Colony formation assays

GBM cells were treated with senescent astrocyte supernatant with or without RA for 24 h, and then GBM cells were cultured in 96-well plates overnight for attachment. Then 100 μL per well FBS-free medium with 10% CCK8 (HY-K0301, MedChemExpress) was incubated the cells for 1 h at 37°C. Microplate reader (BioTek, USA) was adopted to detect the OD values at 450 nm. These steps mentioned above were repeated at 24 h, 48 h, 72 h, 96 h, and 120 h. Regarding colony formation experiment, GBM cells were seeded in 6-well plates and allowed to grow until visible colonies formed. Then we used 4% PFA to fix clones 15 min, 1% crystal violet to stain clones 20 min, and counted the number of clones (>50 cells).

Transwell migration and Wound healing assays

GBM cells were treated as previously reported, then GBM cells were seeded in the upper Transwell chamber with 200 μL FBS-free medium, and 500 μL medium containing 20% FBS was added to the lower chambers. After 24 h incubation, the cells that migrated through membranes were fixed with 4% PFA, stained with 1% crystal violet and counted under light microscope equipped with a ×20 objective. For wound healing assays, GBM cells were cultured in 24-well plates when the cells were grown to 100% density, then utilized 10 μL pipette tips to create scratch wounds. The cells were cultured in DMEM medium without FBS. Images of wounds were captured at 24 h, 48 h, and 72 h, and the area of wound closure was measured at three randomly selected locations in each image using ImageJ, and the average value was taken. Migration rate (%) = $(A_0 - A_t)/A_0 \times 100$, where A_0 is the initial wound area, and A_t represents the wound area at 24 h, 48 h or 72 h.

Figure 1H

P16 16KD

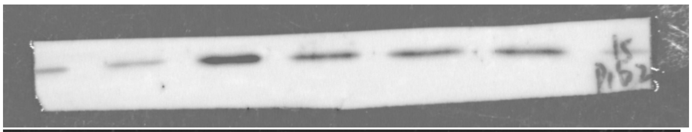

15

GAPDH 36KD

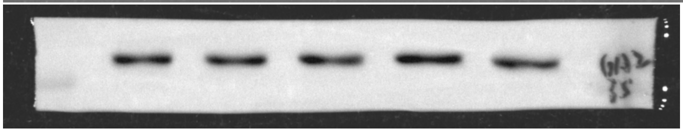

35

10Gy	-	+	+	+	+
RA（μM）	-	-	0.01	0.1	1

TOTAL

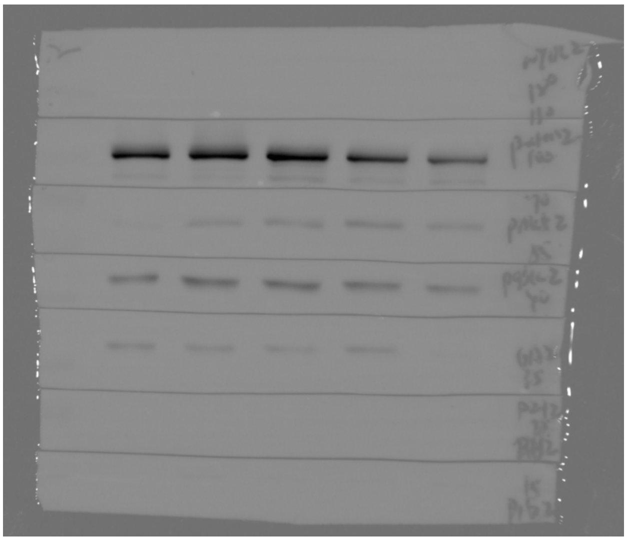

γH2AX 15KD

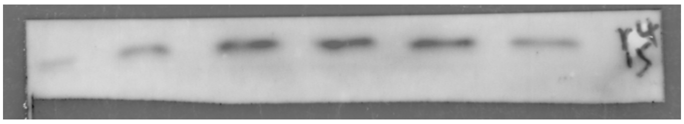

15

GAPDH 36KD

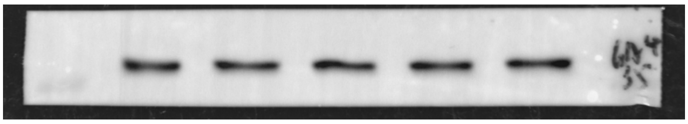

35

10Gy	-	+	+	+	+
RA（μM）	-	-	0.01	0.1	1

TOTAL

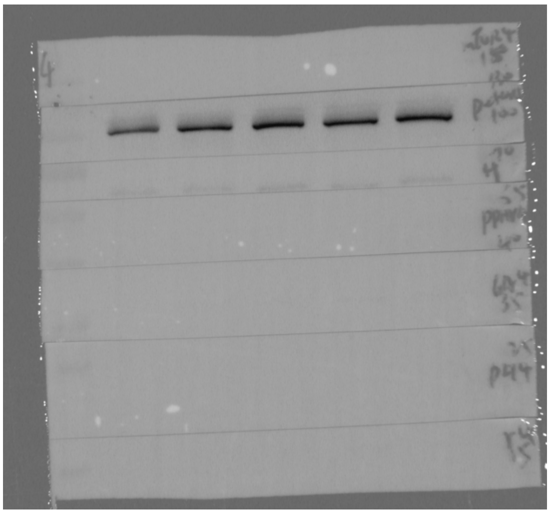

Figure 3H

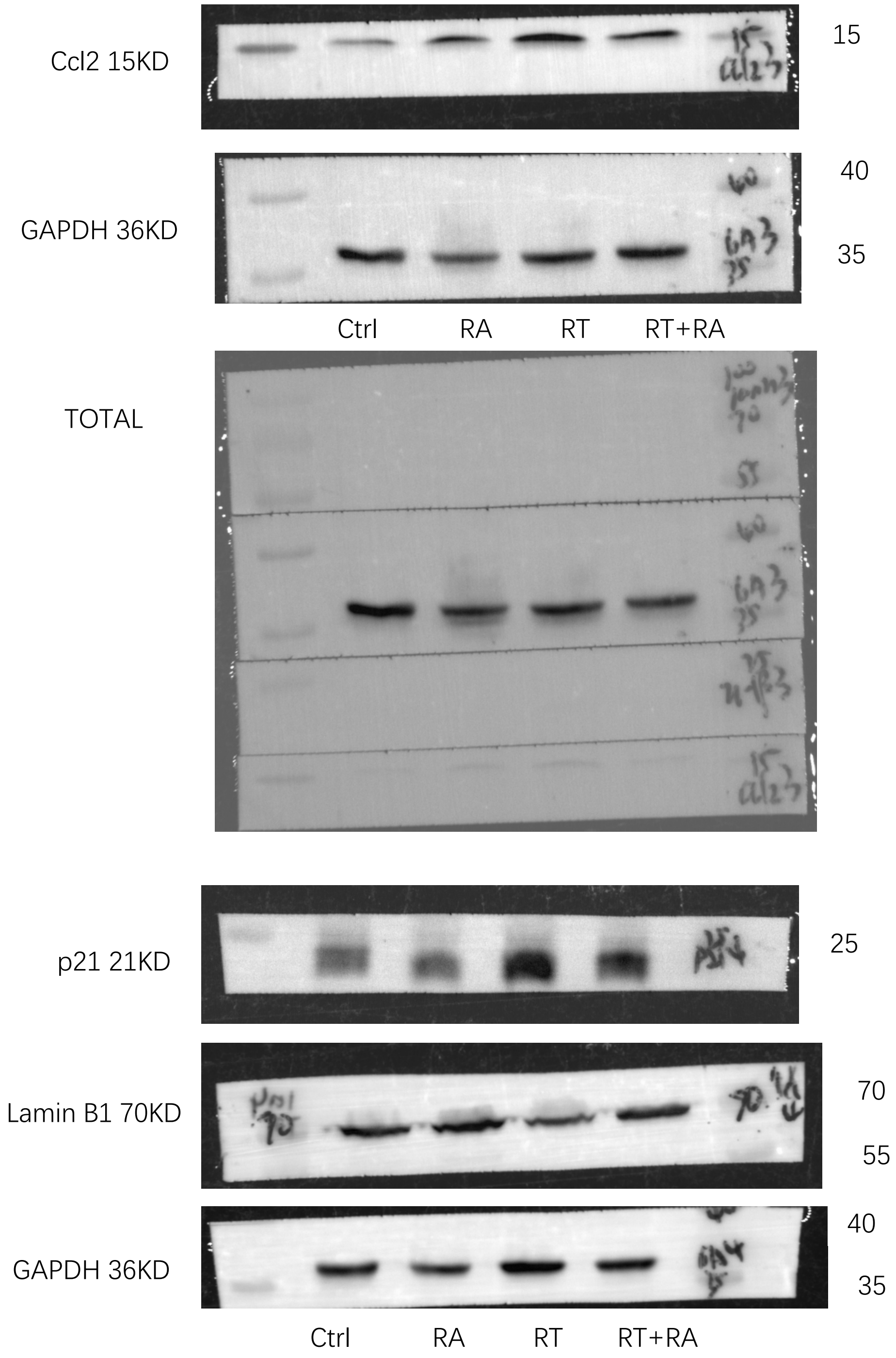

Ccl2 15KD
15
GAPDH 36KD
40
35
Ctrl
RA
RT
RT+RA
TOTAL
p21 21KD
25
Lamin B1 70KD
70
55
GAPDH 36KD
40
35
Ctrl
RA
RT
RT+RA

TOTAL

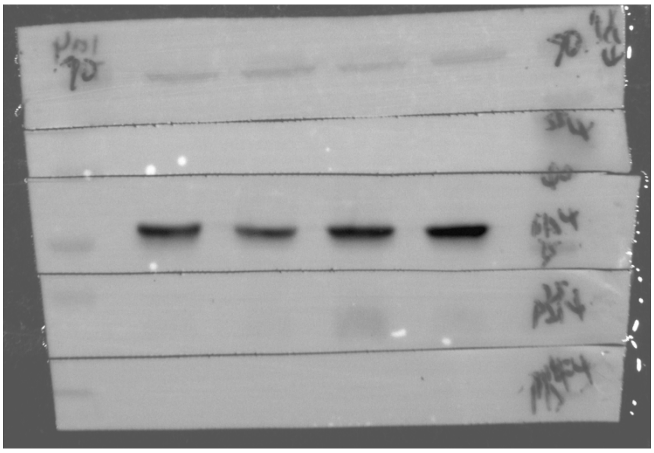

IL6 23KD

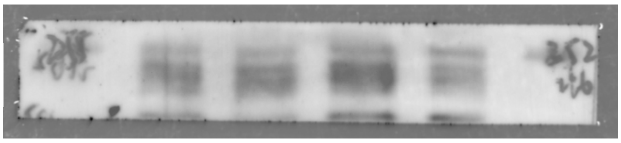

25

GAPDH 36KD

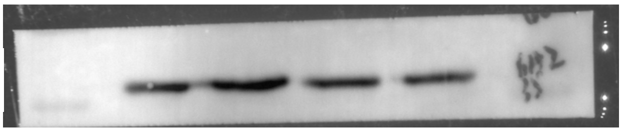

40

35

Ctrl RA RT RT+RA

IL1B 23KD

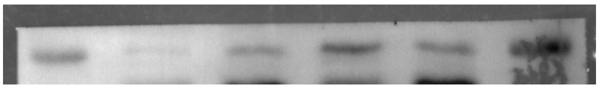

25

GAPDH 36KD

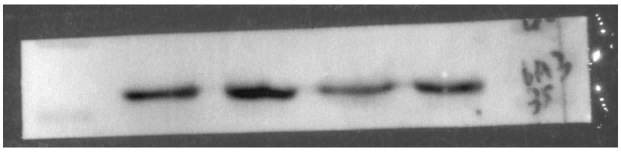

40

35

Ctrl RA RT RT+RA

Figure 4J

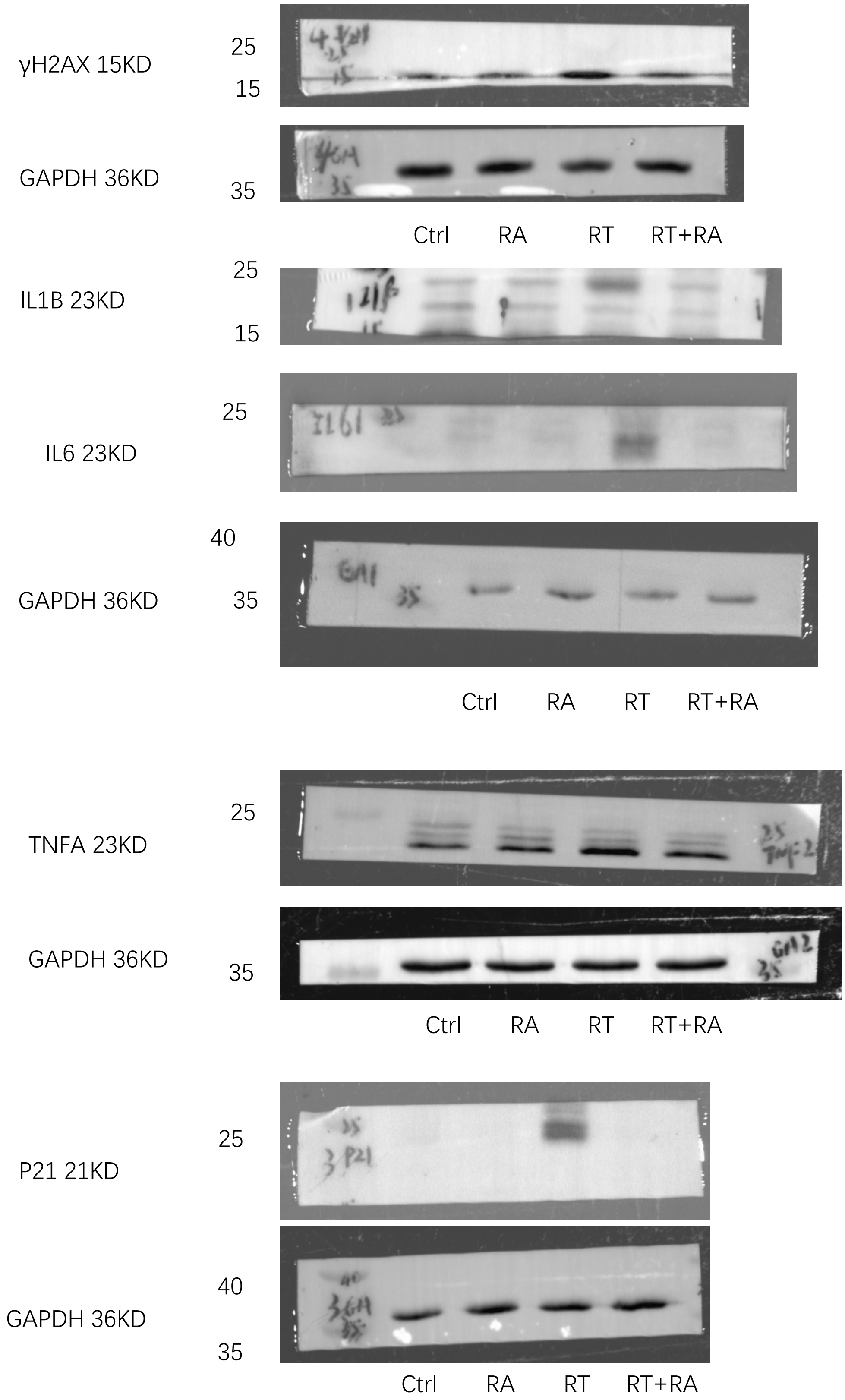
γH2AX 15KD
25
15
GAPDH 36KD
35
Ctrl
RA
RT
RT+RA
IL1B 23KD
25
15
IL6 23KD
25
GAPDH 36KD
40
35
Ctrl
RA
RT
RT+RA
TNFA 23KD
25
GAPDH 36KD
35
Ctrl
RA
RT
RT+RA
P21 21KD
25
GAPDH 36KD
40
35
Ctrl
RA
RT
RT+RA

Figure 5A

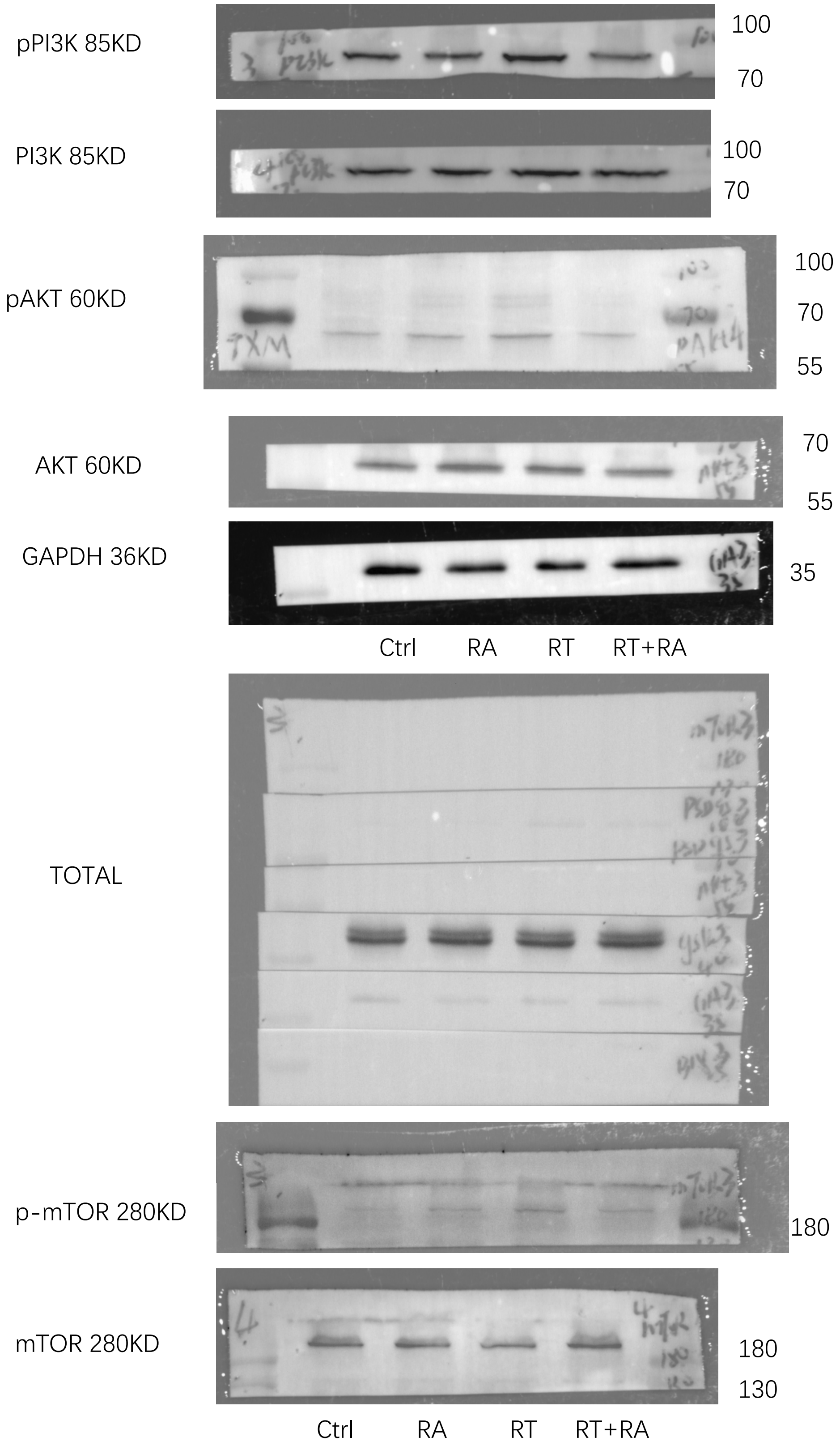
pPI3K 85KD
100
70
PI3K 85KD
100
70
pAKT 60KD
100
70
55
AKT 60KD
70
55
GAPDH 36KD
35
Ctrl
RA
RT
RT+RA
TOTAL
p-mTOR 280KD
180
mTOR 280KD
180
130
Ctrl
RA
RT
RT+RA

PPARr 54KD

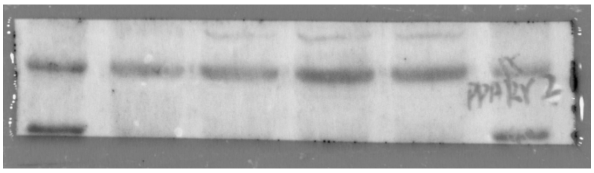

55

40

GAPDH 36KD

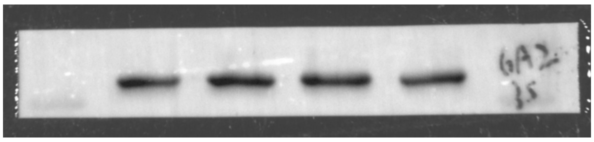

35

Ctrl RA RT RT+RA

TOTAL

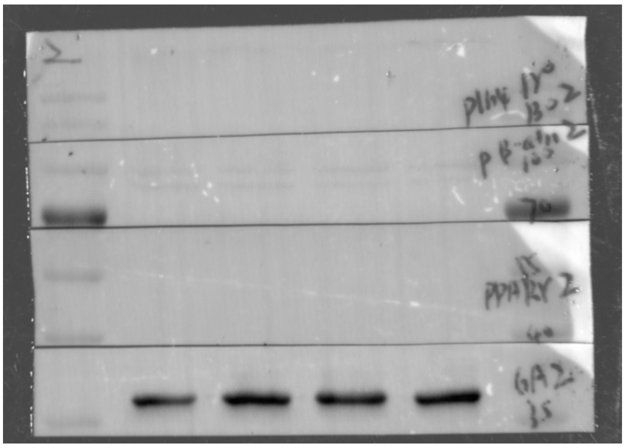

Plin4 134 KD

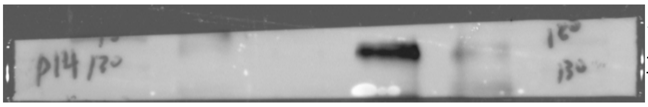

180

130

GAPDH 36KD

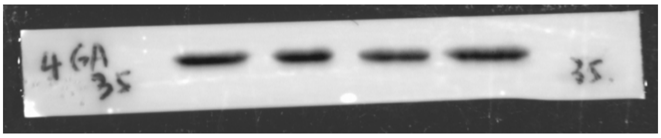

35

Ctrl RA RT RT+RA

TOTAL

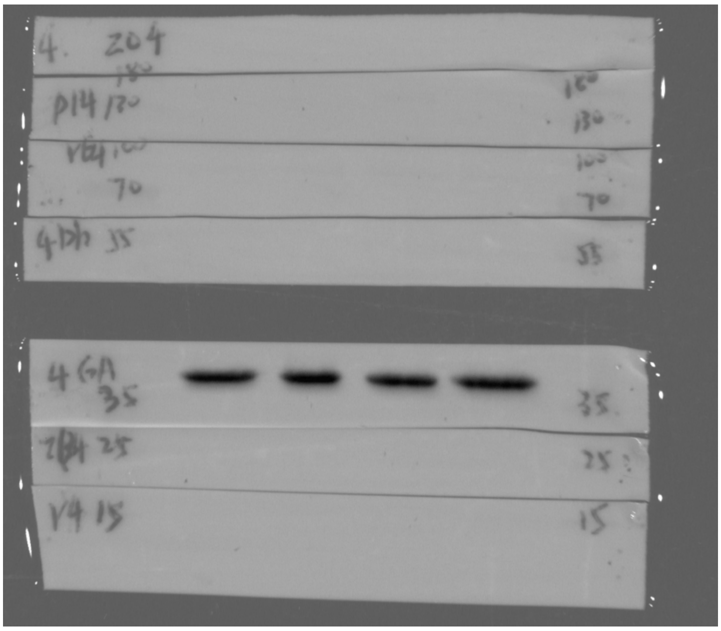

Supplemental Figure 1D

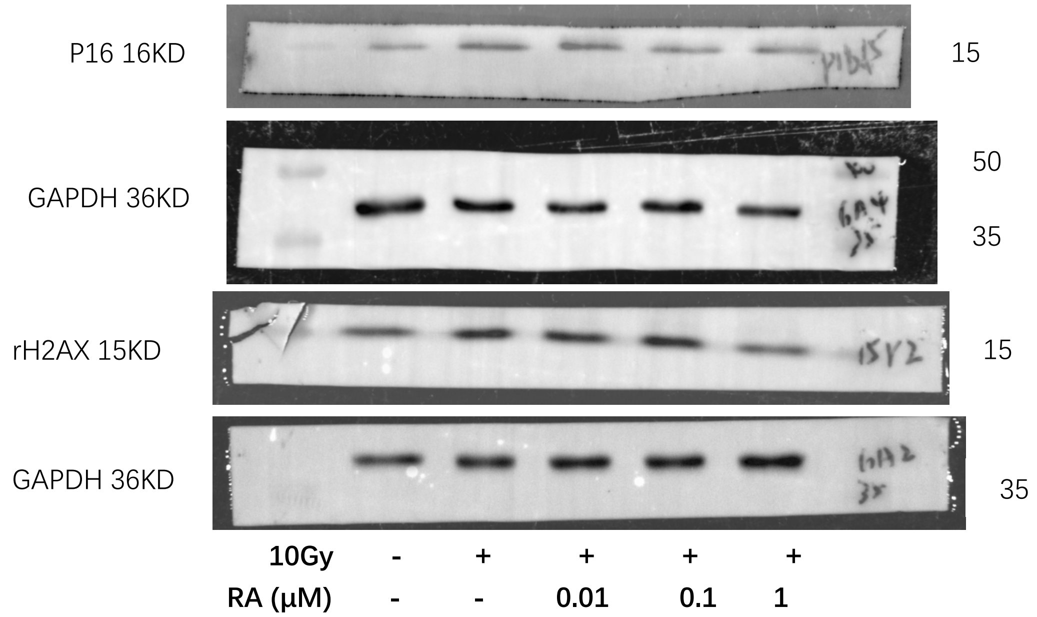

Supplemental Figure 3F

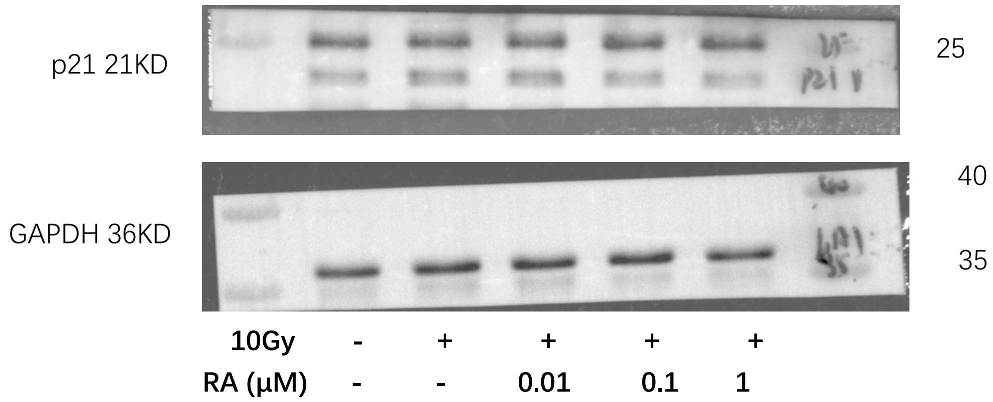

Supplemental Figure 4M

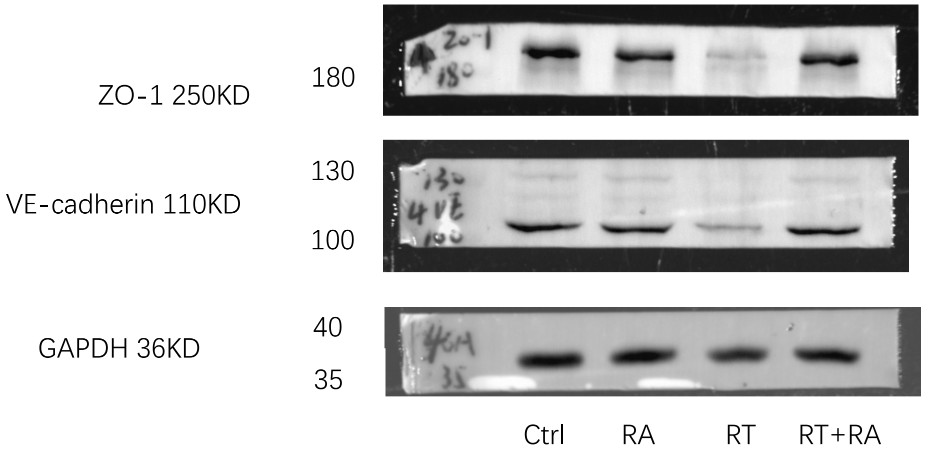

Supplemental Figure 5D

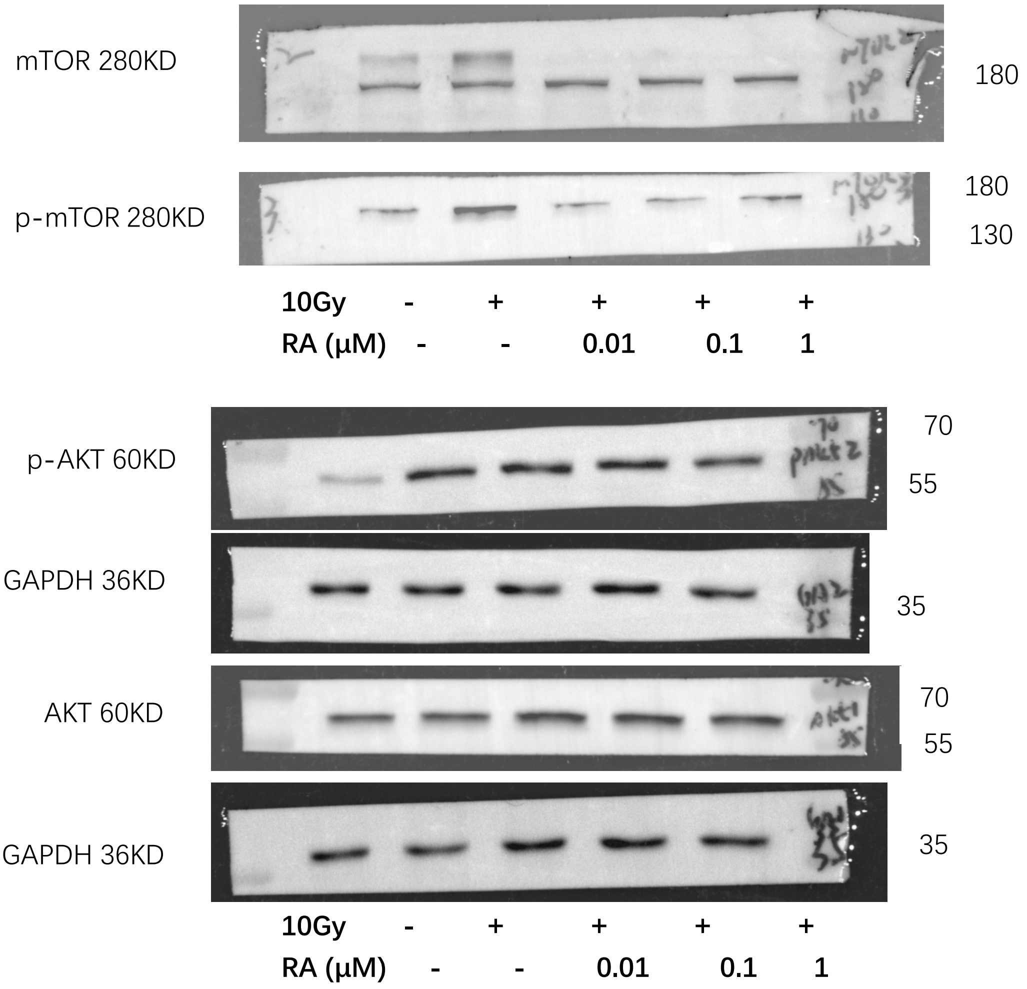

TOTAL

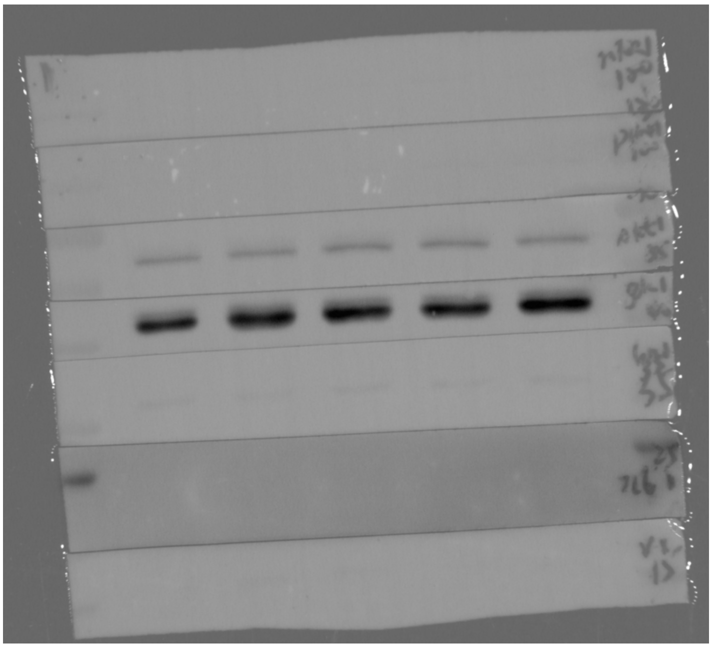

Plin4 100KD

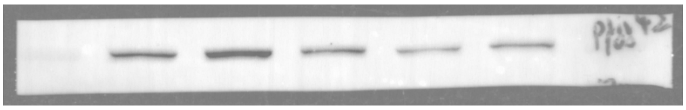

100

GAPDH 36KD

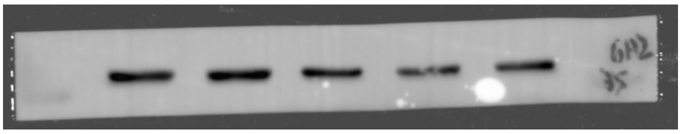

35

TOTAL

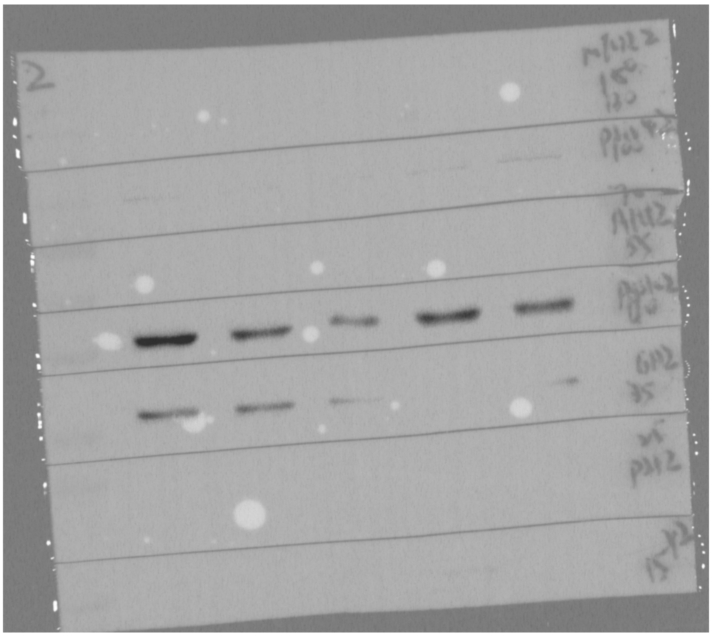

PPARr 54KD

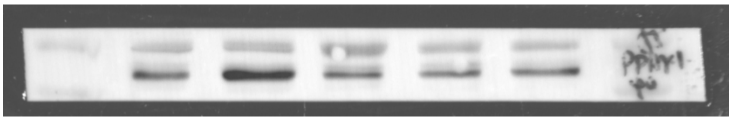

55

40

GAPDH 36KD

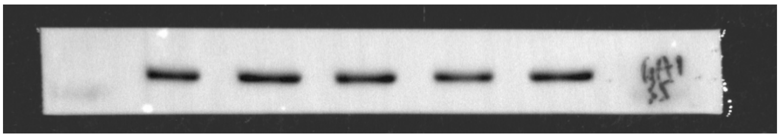

35

**10Gy**	**-**	**+**	**+**	**+**	**+**
**RA (μM)**	**-**	**-**	**0.01**	**0.1**	**1**

TOTAL

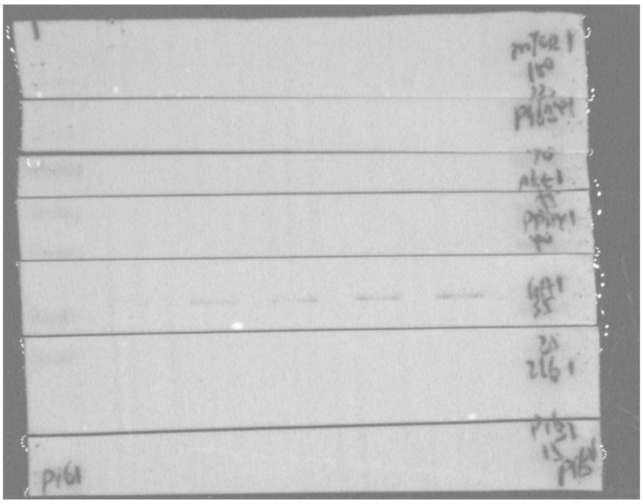

Supplemental Figure 5E

p-mTOR 280KD
mTOR 280KD
GAPDH 36KD

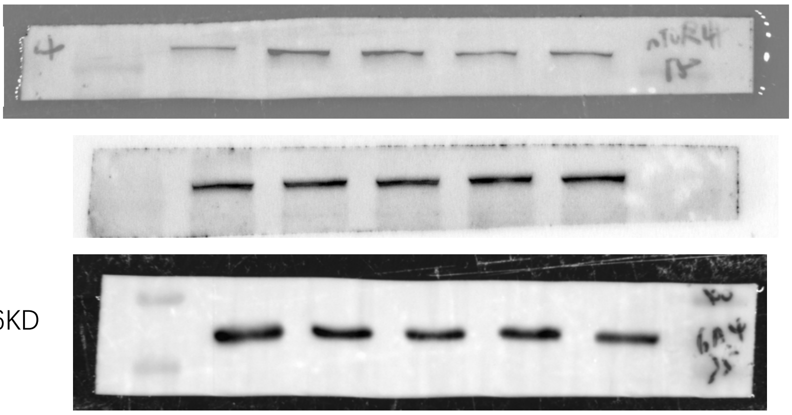

280
280
40
35

10Gy	-	+	+	+	+
RA (μM)	-	-	0.01	0.1	1

TOTAL

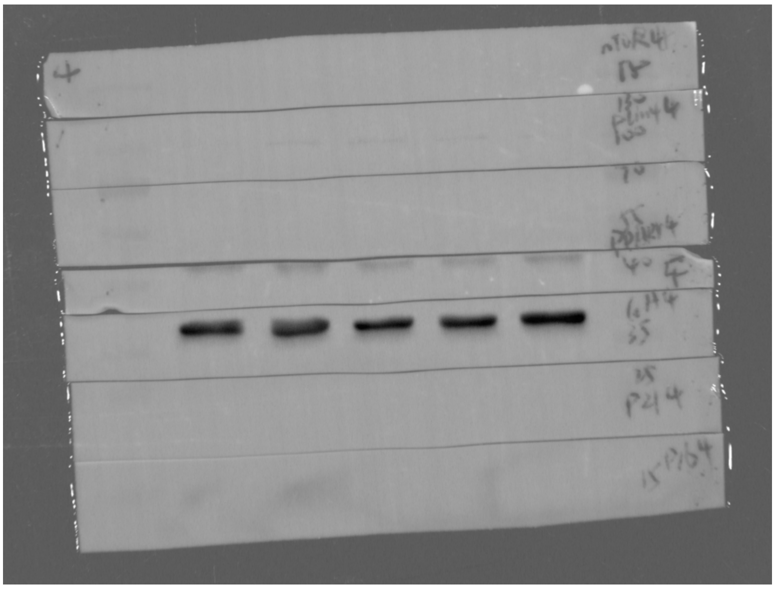

p-AKT 60KD
AKT 60KD
GAPDH 36KD

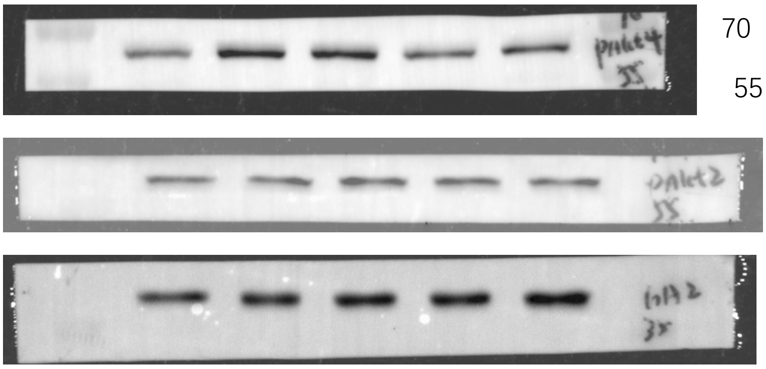

70
55
70
55
35

10Gy	-	+	+	+	+
RA (μM)	-	-	0.01	0.1	1

TOTAL

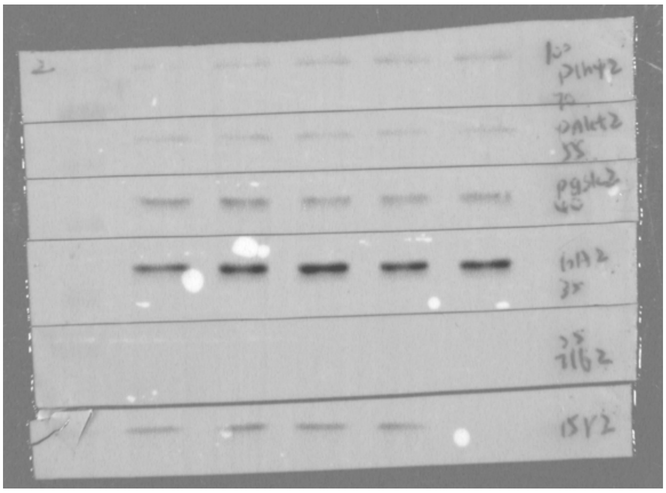

Plin4 100KD

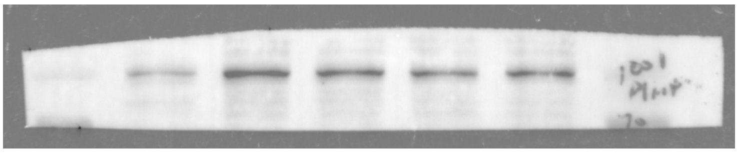

100

70

GAPDH 36KD

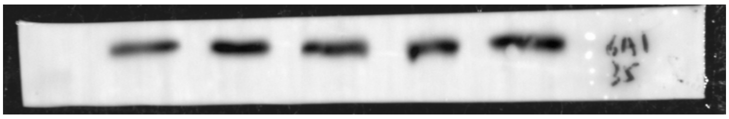

35

10Gy	-	+	+	+	+
RA (μM)	-	-	0.01	0.1	1

TOTAL

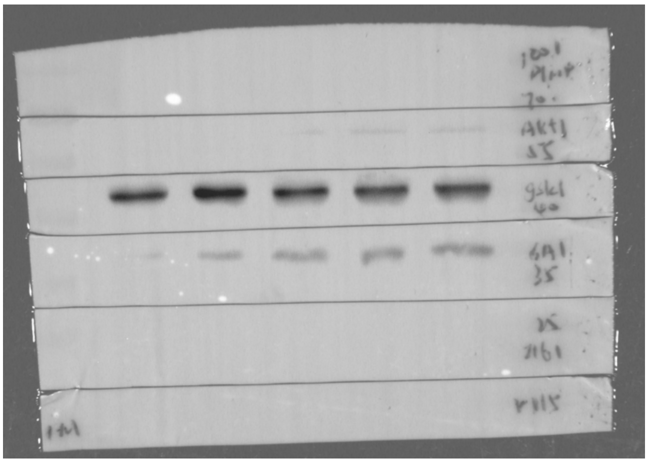

PPARr 54KD

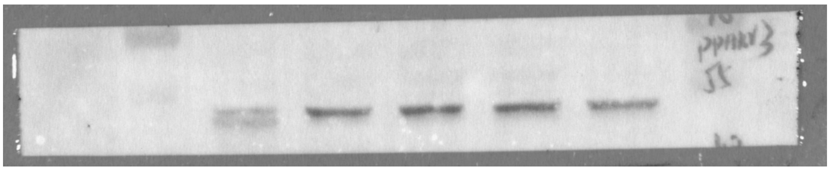

70

55

40

10Gy	-	+	+	+	+
RA (μM)	-	-	0.01	0.1	1
