# Supplementary material for: All-trans retinoic acid inhibits glioblastoma progression and attenuates radiation-induced brain injury
Source: JCI Insight. 2024 Nov 8;9(21):e179530. doi: 10.1172/jci.insight.179530 (PMC11601587; doi:10.1172/jci.insight.179530)
Supplement: Unedited blot and gel images [file jciinsight-9-179530-s020.pdf]

Figure 1H

P16 16KD

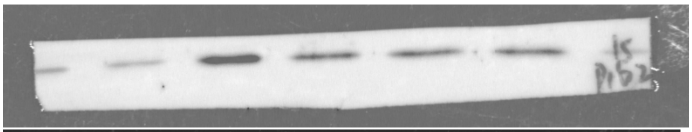

15

GAPDH 36KD

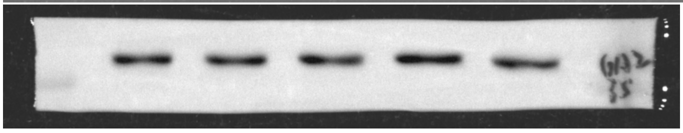

35

10Gy	-	+	+	+	+
RA（μM）	-	-	0.01	0.1	1

TOTAL

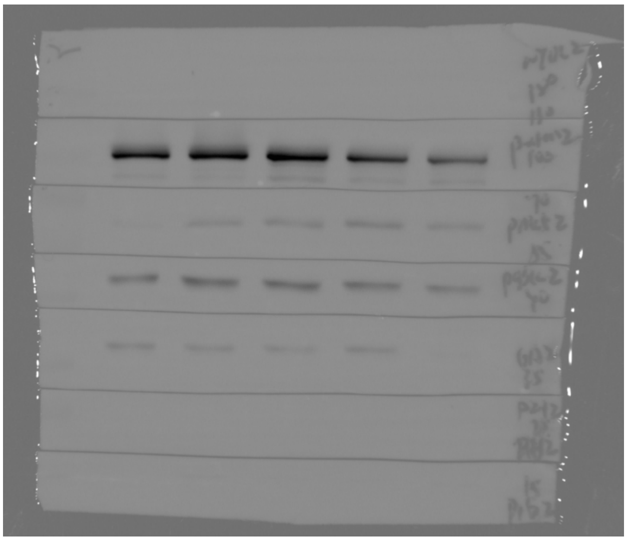

γH2AX 15KD

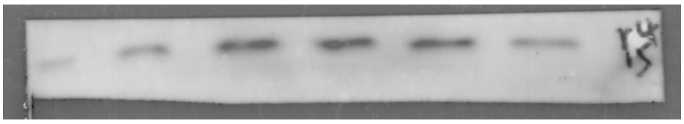

15

GAPDH 36KD

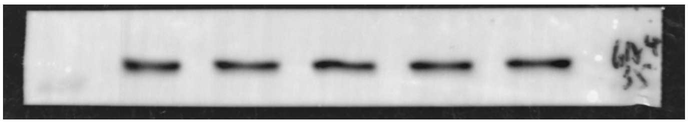

35

10Gy	-	+	+	+	+
RA（μM）	-	-	0.01	0.1	1

TOTAL

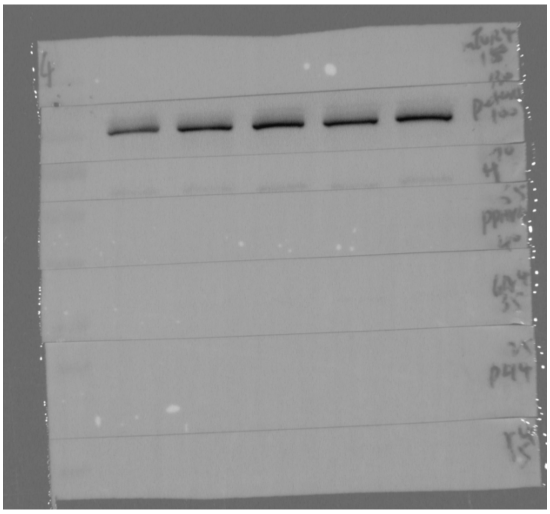

Figure 3H

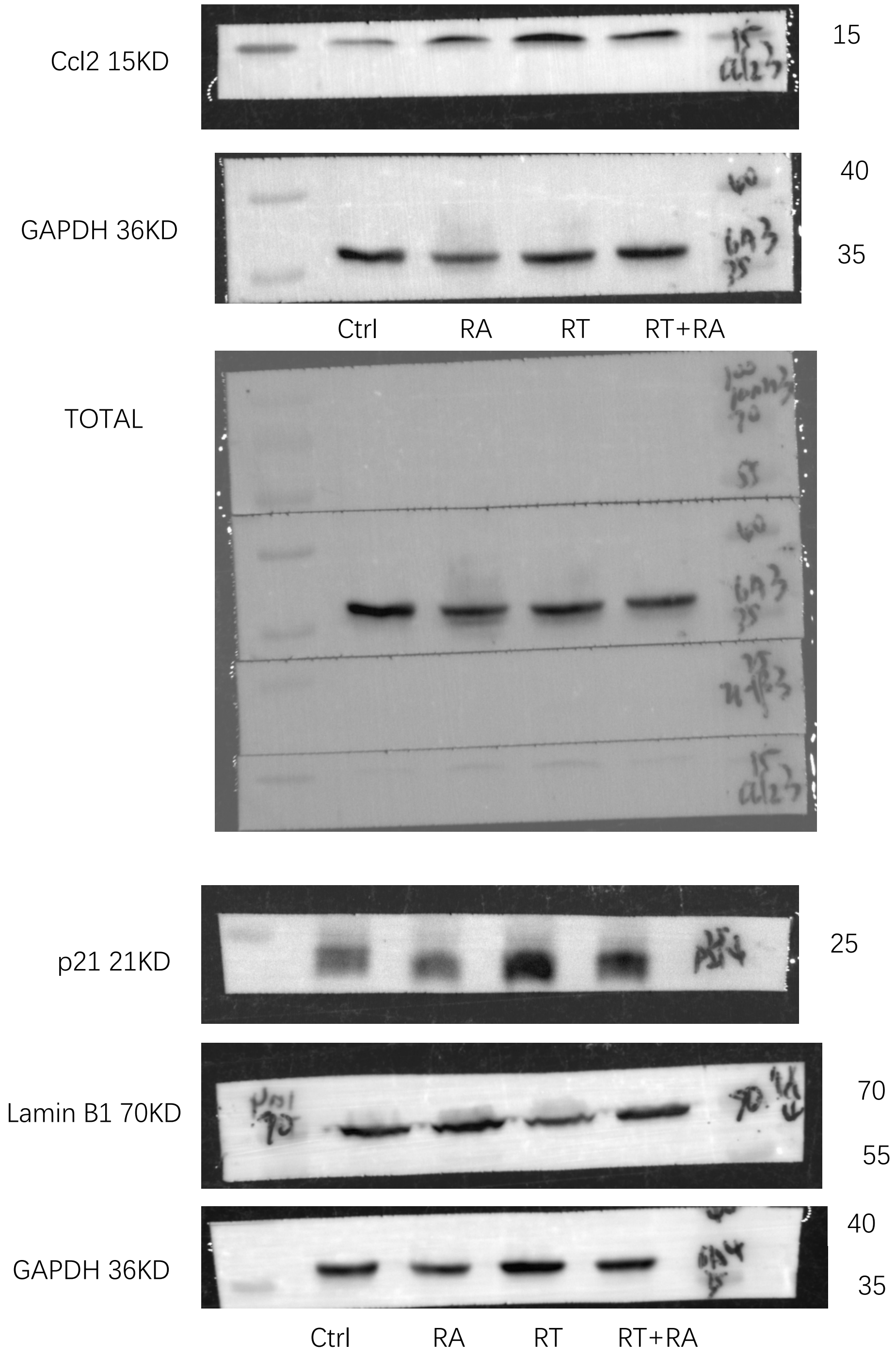
Ccl2 15KD
15
GAPDH 36KD
40
35
Ctrl
RA
RT
RT+RA
TOTAL
p21 21KD
25
Lamin B1 70KD
70
55
GAPDH 36KD
40
35
Ctrl
RA
RT
RT+RA

TOTAL

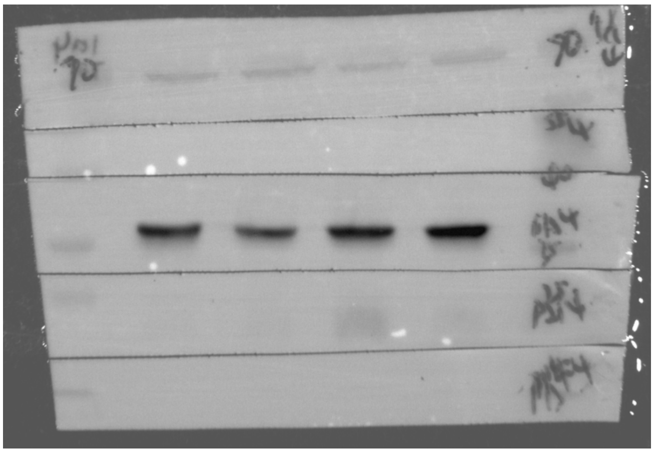

IL6 23KD

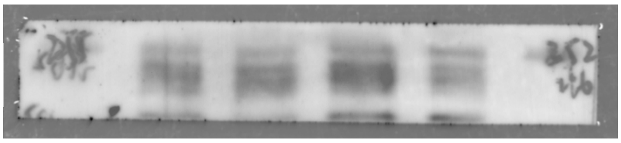

25

GAPDH 36KD

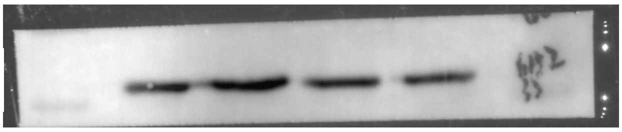

40

35

Ctrl RA RT RT+RA

IL1B 23KD

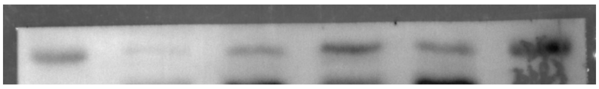

25

GAPDH 36KD

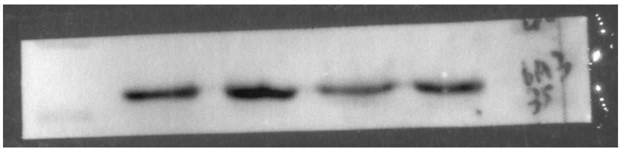

40

35

Ctrl RA RT RT+RA

Figure 4J

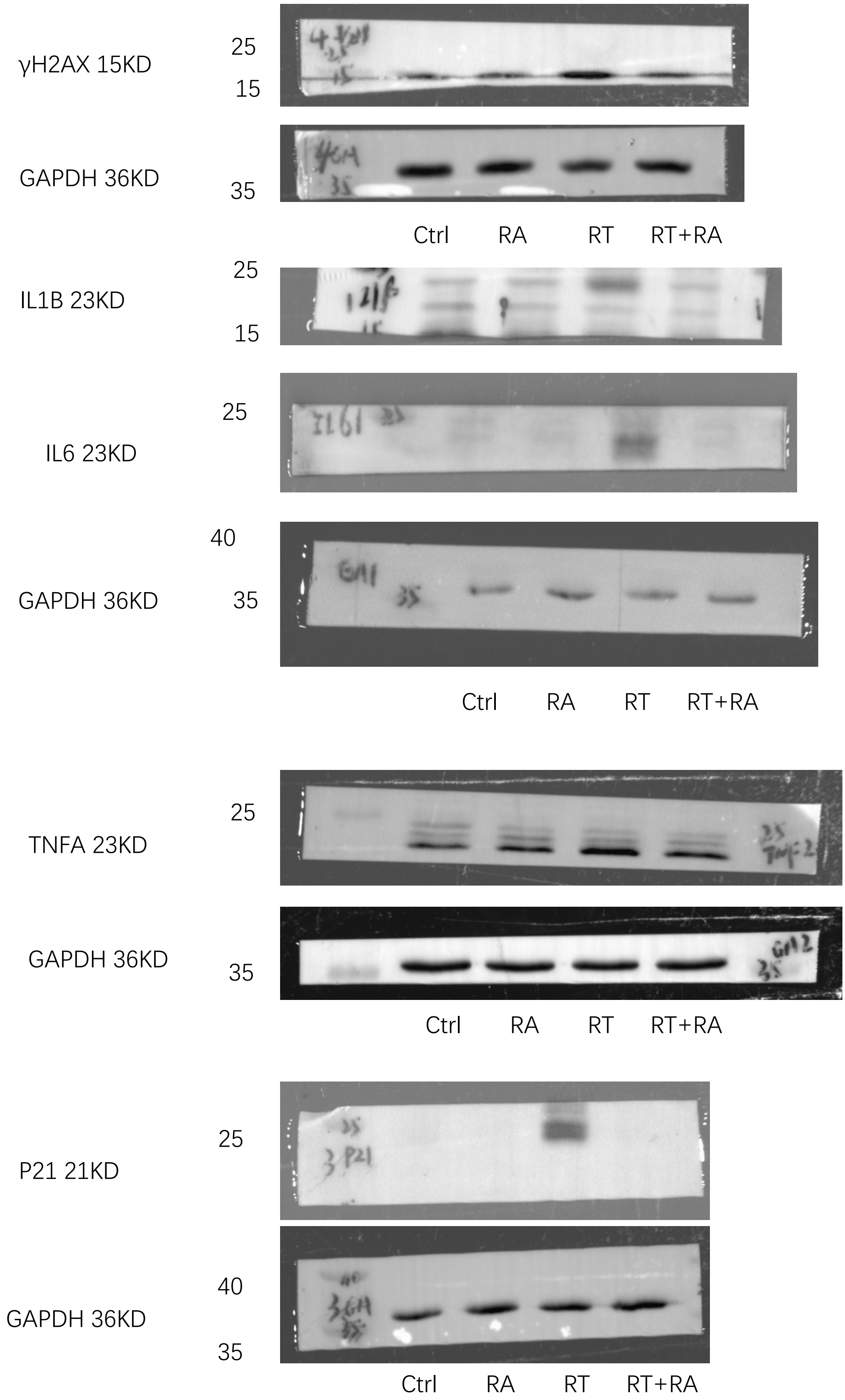
γH2AX 15KD
25
15
GAPDH 36KD
35
Ctrl
RA
RT
RT+RA
IL1B 23KD
25
15
IL6 23KD
25
40
GAPDH 36KD
35
Ctrl
RA
RT
RT+RA
TNFA 23KD
25
GAPDH 36KD
35
Ctrl
RA
RT
RT+RA
P21 21KD
25
40
GAPDH 36KD
35
Ctrl
RA
RT
RT+RA

Figure 5A

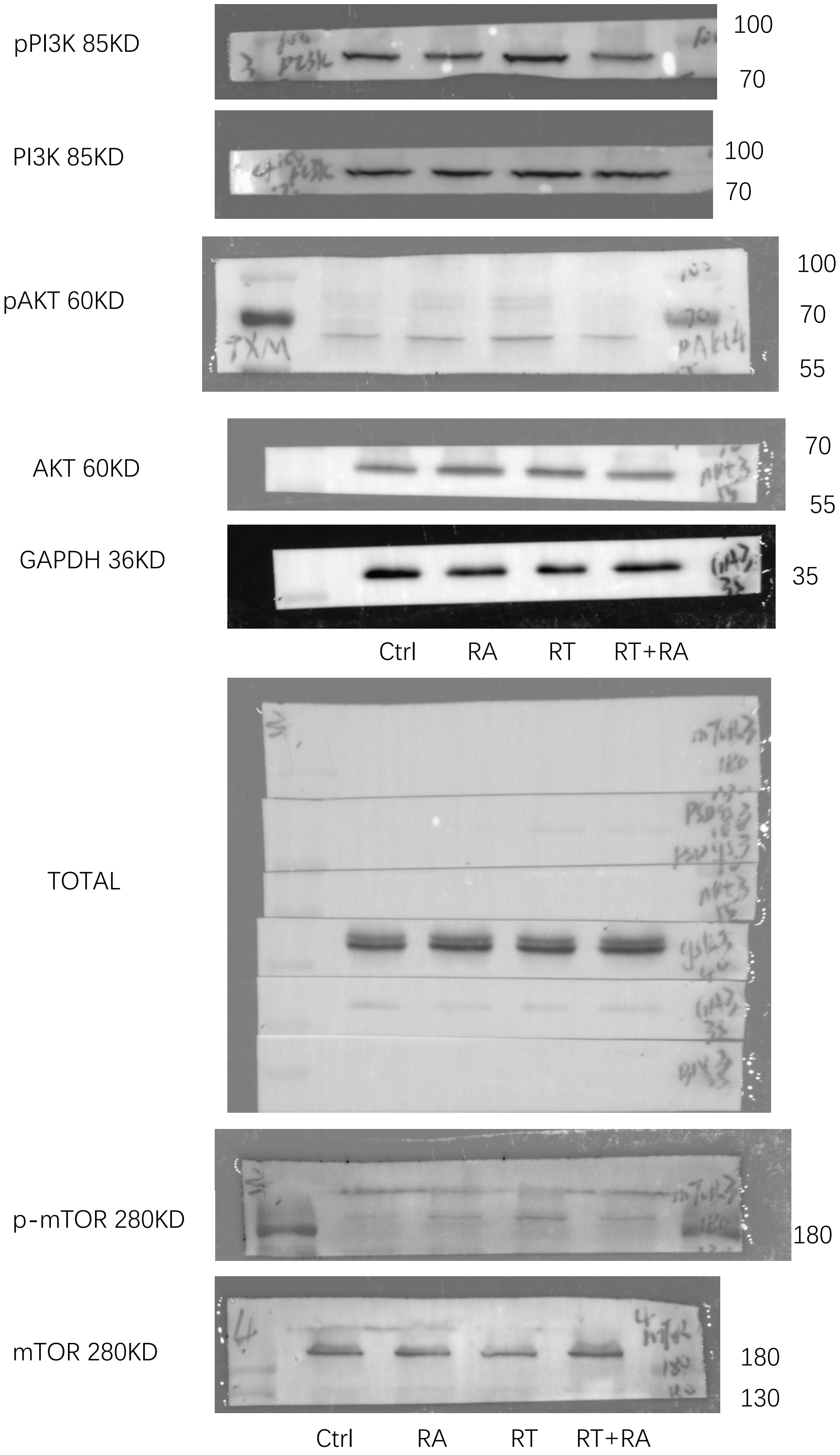
pPI3K 85KD
100
70
PI3K 85KD
100
70
pAKT 60KD
100
70
55
AKT 60KD
70
55
GAPDH 36KD
35
Ctrl
RA
RT
RT+RA
TOTAL
p-mTOR 280KD
180
mTOR 280KD
180
130
Ctrl
RA
RT
RT+RA

PPARr 54KD

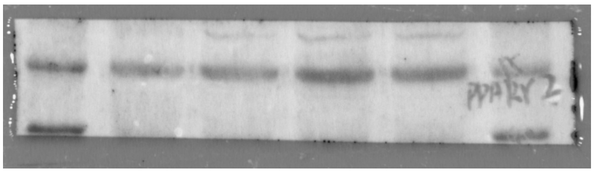

55

40

GAPDH 36KD

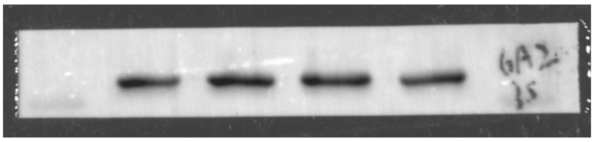

35

Ctrl RA RT RT+RA

TOTAL

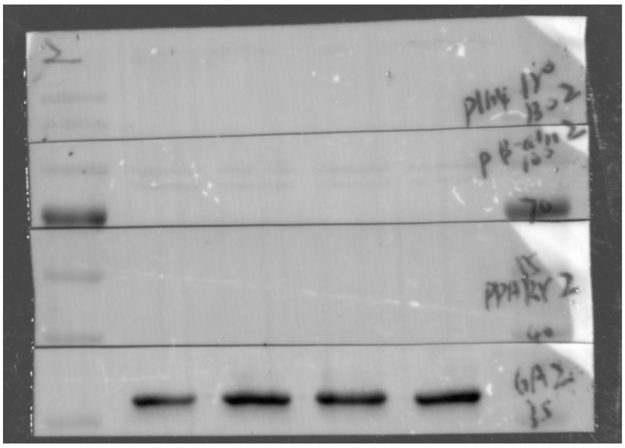

Plin4 134 KD

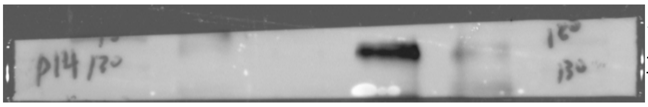

180

130

GAPDH 36KD

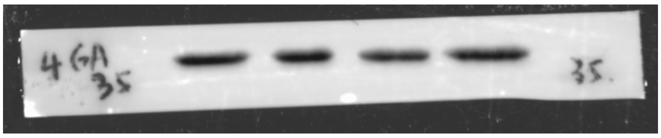

35

Ctrl RA RT RT+RA

TOTAL

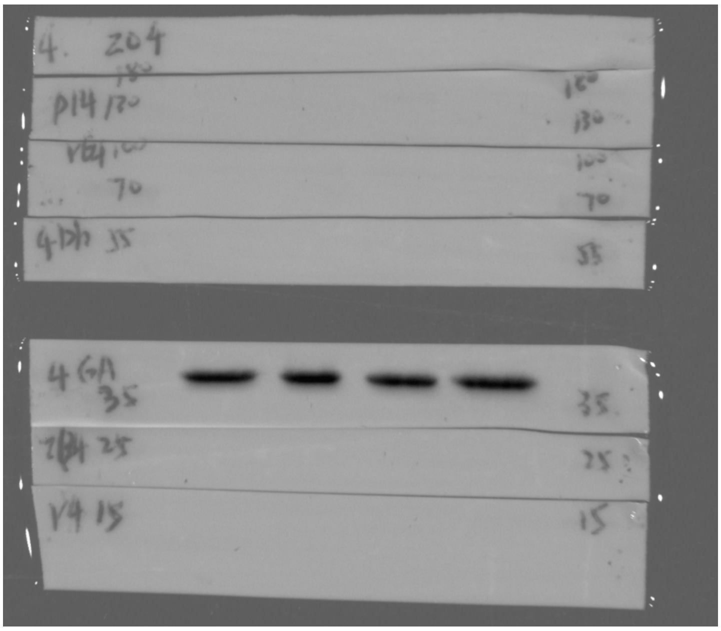

Supplemental Figure 1D

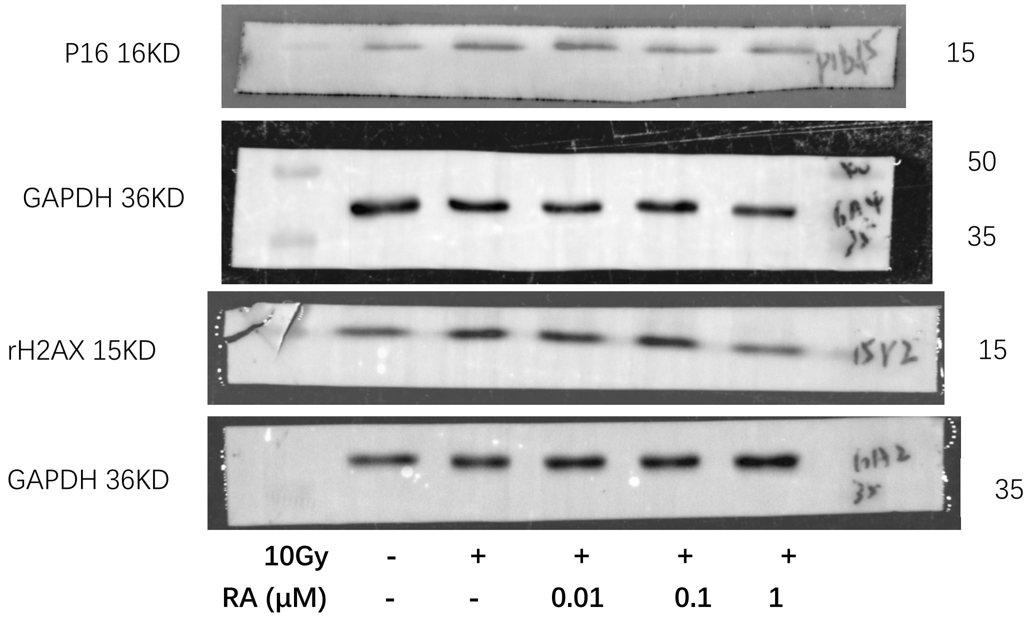

Supplemental Figure 3F

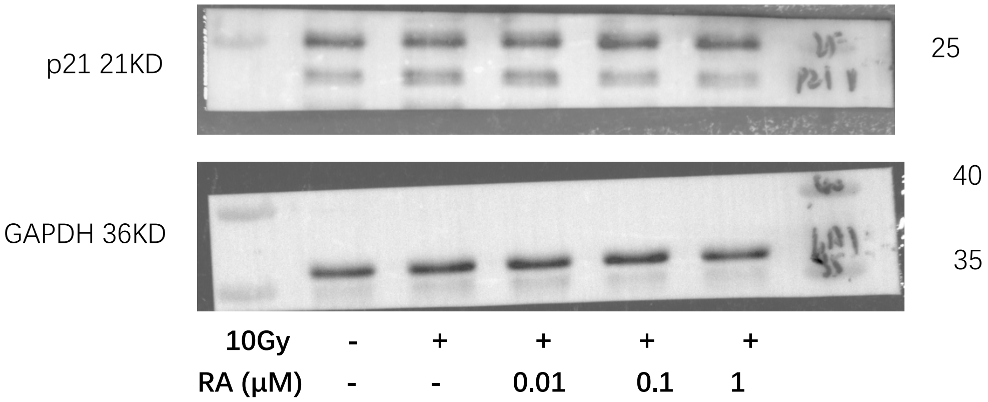

Supplemental Figure 4M

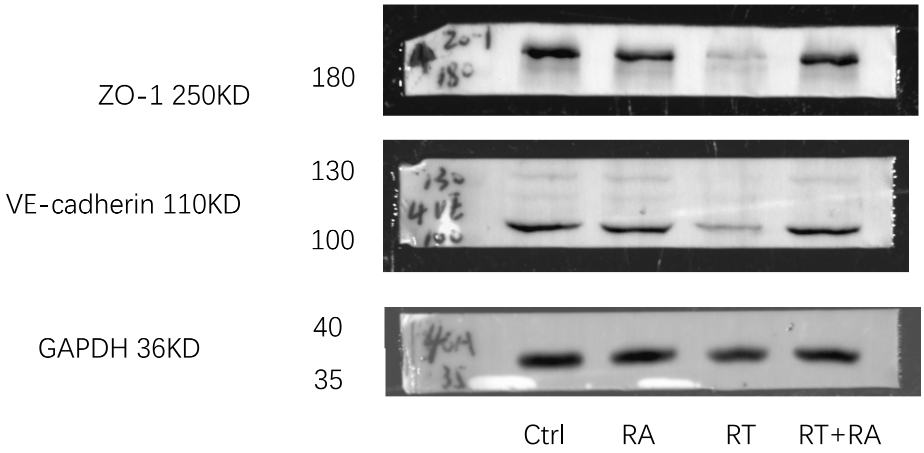

Supplemental Figure 5D

mTOR 280KD

p-mTOR 280KD

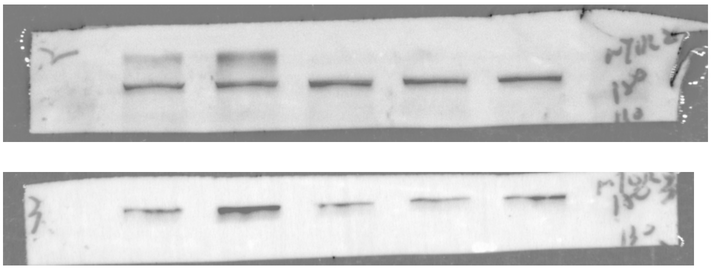

180

180

130

10Gy - + + + +

RA (μM) - - 0.01 0.1 1

p-AKT 60KD

GAPDH 36KD

AKT 60KD

GAPDH 36KD

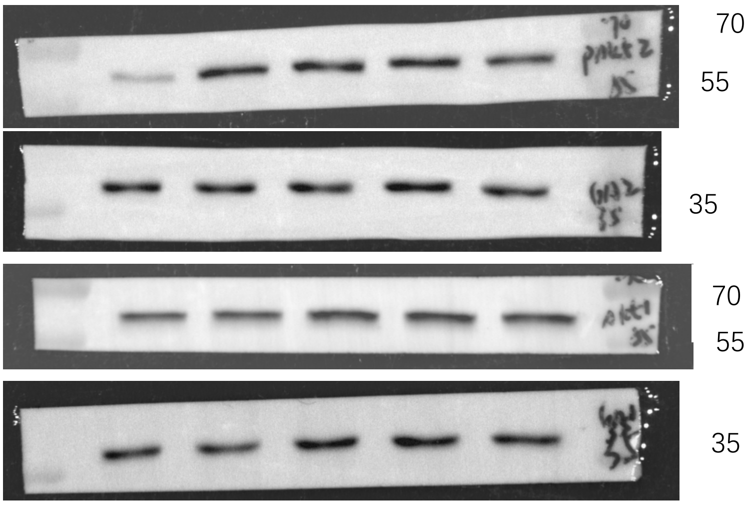

70

55

35

70

55

35

10Gy - + + + +

RA (μM) - - 0.01 0.1 1

TOTAL

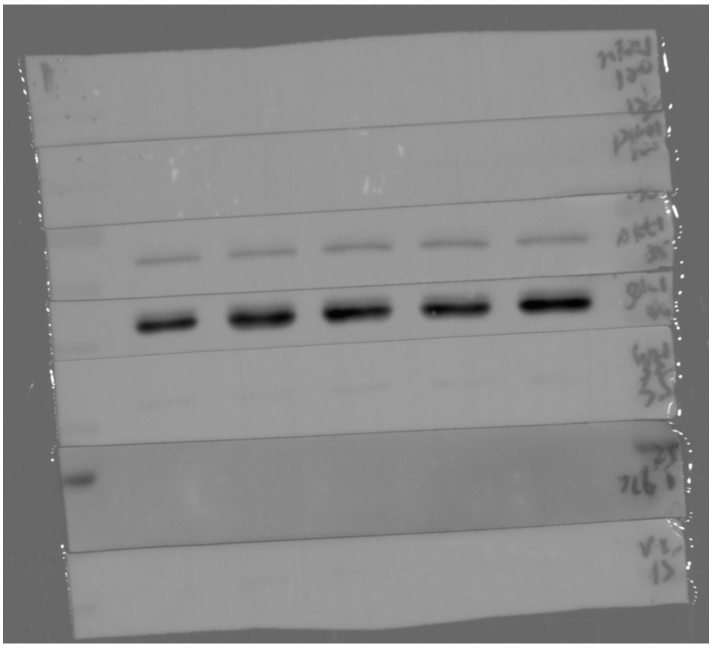

Plin4 100KD

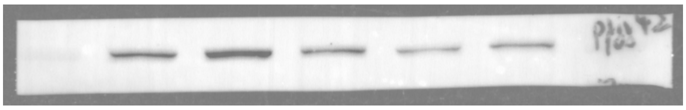

100

GAPDH 36KD

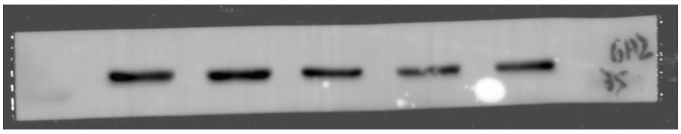

35

TOTAL

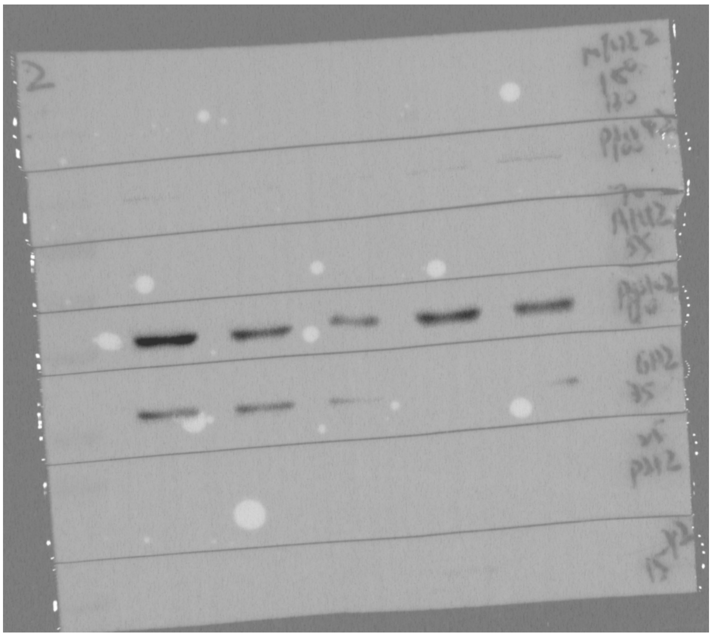

PPARr 54KD

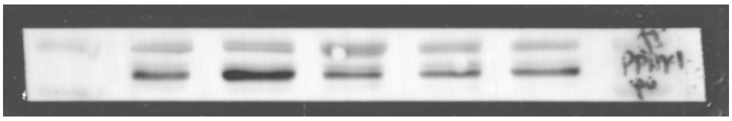

55

40

GAPDH 36KD

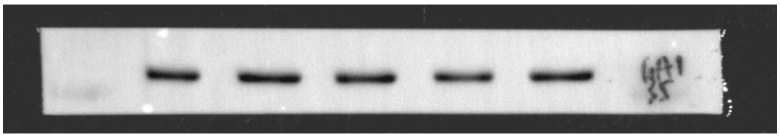

35

**10Gy**	**-**	**+**	**+**	**+**	**+**
**RA (μM)**	**-**	**-**	**0.01**	**0.1**	**1**

TOTAL

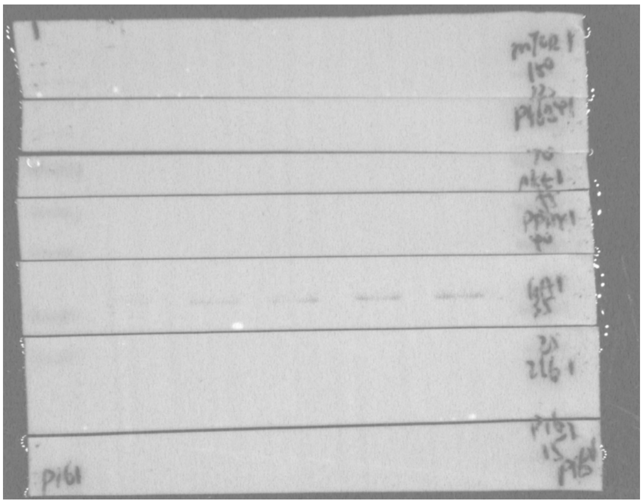

Supplemental Figure 5E

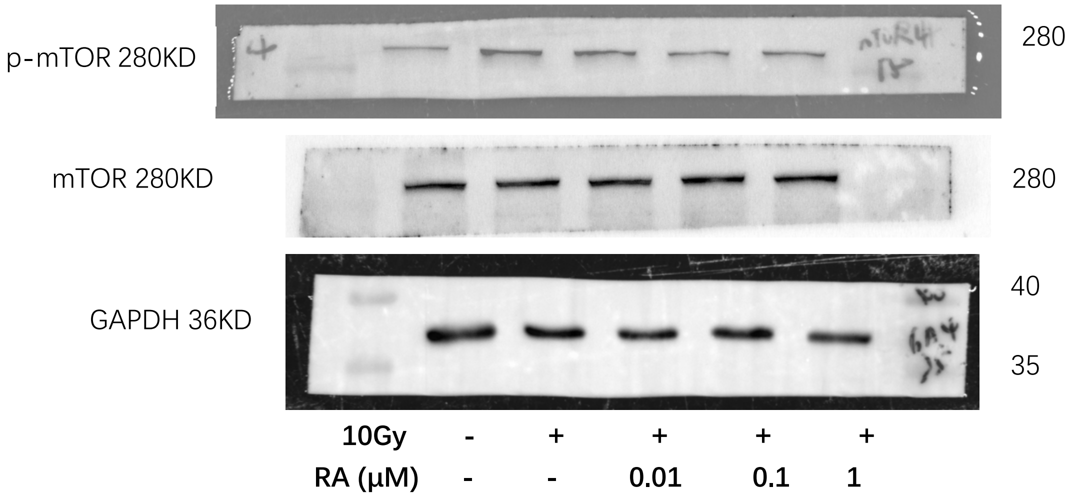

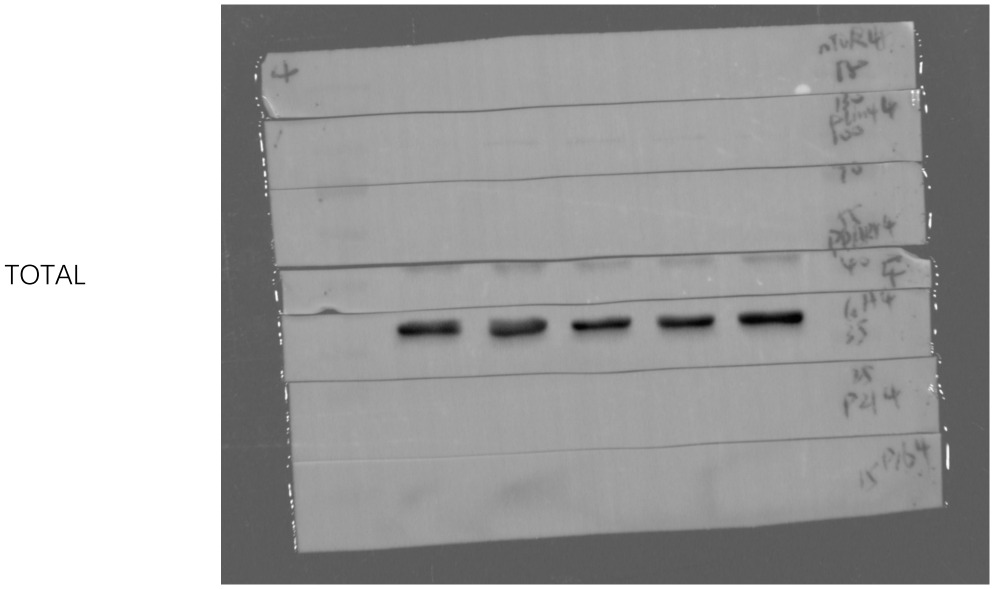

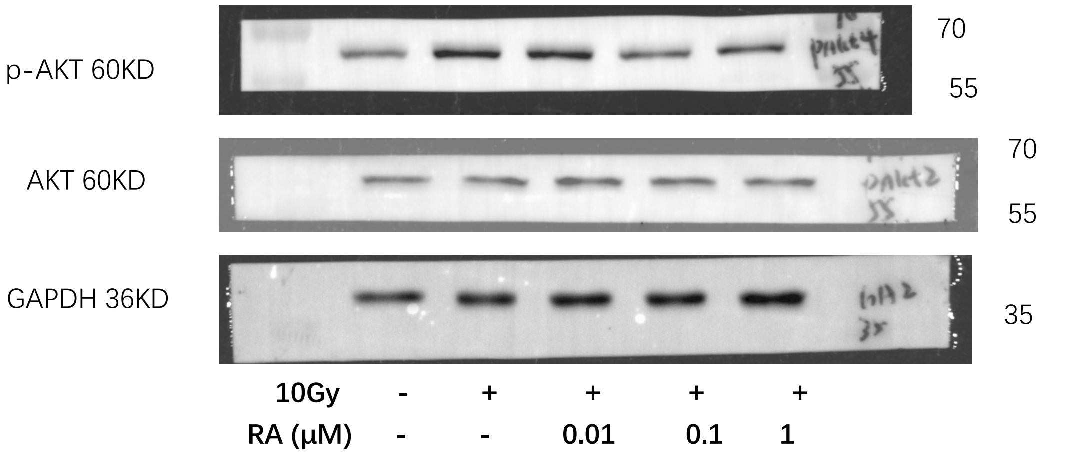

TOTAL

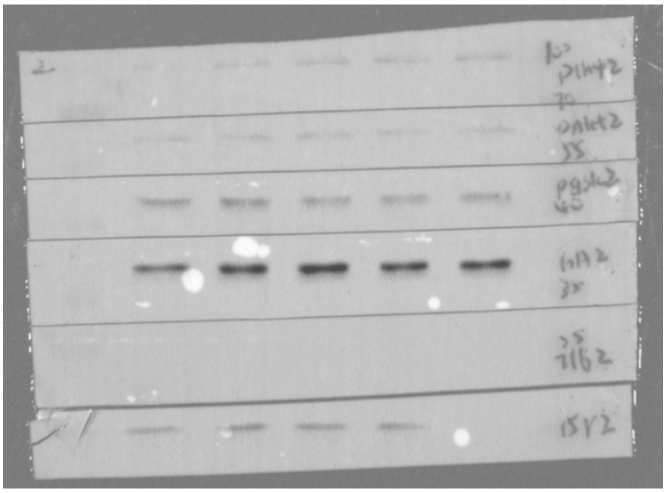

Plin4 100KD

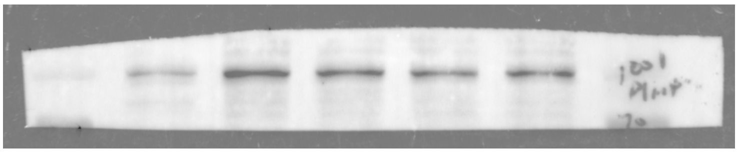

100

70

GAPDH 36KD

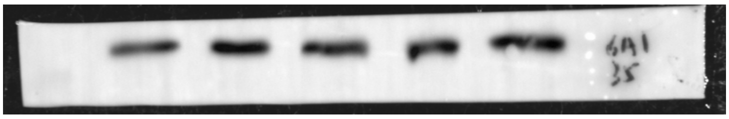

35

10Gy	-	+	+	+	+
RA (μM)	-	-	0.01	0.1	1

TOTAL

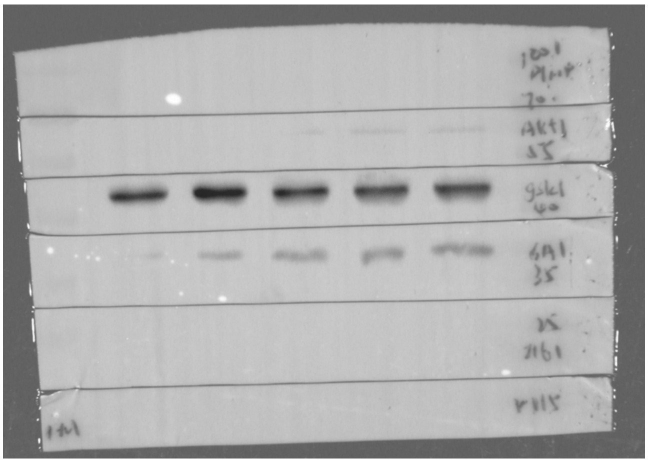

PPARr 54KD

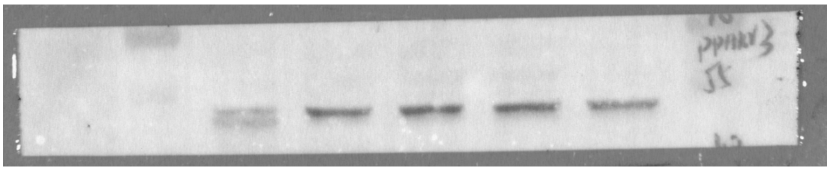

70

55

40

10Gy	-	+	+	+	+
RA (μM)	-	-	0.01	0.1	1
